# Plasma Microbial Cell-Free DNA Sequencing Technology for the Diagnosis of Sepsis in the ICU

**DOI:** 10.3389/fmolb.2021.659390

**Published:** 2021-05-28

**Authors:** Lili Wang, Wenzheng Guo, Hui Shen, Jian Guo, Donghua Wen, Yuetian Yu, Wenjuan Wu

**Affiliations:** ^1^ Department of Laboratory Medicine, Shanghai East Hospital, Tongji University School of Medicine, Shanghai, China; ^2^ Department of Critical Care Medicine, Ren Ji Hospital, Shanghai Jiao Tong University School of Medicine, Shanghai, China

**Keywords:** microbial cell-free DNA, sequencing, culture, diagnosis, sepsis, intensive care unit

## Abstract

Sepsis is a common life-threatening disease in the intensive care unit (ICU) that is usually treated empirically without pathogen identification. As a non-invasive and high-throughput technology, plasma microbial cell-free DNA (mcfDNA) sequencing can detect unknown pathogens independent of previous clinical or laboratory information. In this study, a total of 199 cases suspected of bloodstream infection (BSI) from January 2020 to June 2020 were collected, and potential pathogens were detected by simultaneous blood culture and plasma mcfDNA sequencing. Other clinical microbiological assays were performed within 7 days of plasma mcfDNA sequencing, including smear, culture of samples taken from relevant infected sites, and β-D-glucan/galactomannan (BDG/GM) tests, among others. The diagnoses were classified as sepsis [94 (47.2%)], non-sepsis [87 (43.7%)], and non-infectious disease [18 (9.0%)]. The sensitivity and specificity of plasma mcfDNA sequencing for diagnosing sepsis were 68.1 and 63.2%, respectively, which were significantly better than those of blood culture, especially for the common bacteria that cause hospital-acquired infection, namely, *Acinetobacter baumannii* (*p* < 0.01) and *Klebsiella pneumoniae* (*p* < 0.01), and DNA viruses (plasma mcfDNA sequencing only, *p* < 0.01). However, there was no significant difference in the rate of positivity between plasma mcfDNA sequencing and blood culture for antibiotic-non-exposed cases (43.6 vs. 30.9%, *p* = 0.17). In the non-sepsis group, 44.8% of cases (13/29) detected only by plasma mcfDNA sequencing showed infections in other parts of the body, such as lower respiratory infection (LRI), intra-abdominal infection (IAI) and central nervous system infection (CNSI). For some common pathogens (not including anaerobes), turnaround time (TAT) 3 (TAT from the initiation of blood sample processing by nucleic acid extraction to the completion of sequencing analysis) was longer than TAT1 (TAT from blood culture bottles in Virtuo to off Virtuo). With disease progression, significant dynamic changes in microbial species were clearly detected by plasma mcfDNA sequencing.

## Introduction

Sepsis is one of the most fatal diseases in the intensive care unit (ICU) and accounts for approximately one-third of deaths during hospitalization (Zhang et al., 2016). Thus, faster diagnosis and initiation of treatment are needed to decrease all-cause mortality, and “HOUR-1 BUNDLE,” which is proposed by Surviving Sepsis Campaign (SSC), is recommended (Levy et al., 2018). Nevertheless, the accuracy of initial empirical antibiotic therapy is unsatisfactory, which leads to unnecessary exposure to broad-spectrum antibiotics. Furthermore, for patients with culture-negative sepsis, accurately identifying pathogens remains a challenge (Thorndike and Kollef, 2020).

Microorganisms isolated in pure culture were once thought to be the most common method of identifying the pathogens that cause sepsis (Rajapaksha et al., 2019). Although some detection methods, such as biochemical phenotype detection and matrix-assisted laser desorption/ionization time-of-flight mass spectrometry (MALDI-TOF MS), have been developed, positive results with culture are only 30–40% (Rajapaksha et al., 2019). Therefore, culture-independent technology is required to guide the rational use of antimicrobial drugs (Rhee et al., 2021).

As a molecular diagnostic method, multiplex polymerase chain reaction (PCR) is widely used in pathogen identification of sepsis but cannot meet the demands of complex clinical samples with unknown etiology because of ambiguous presupposition of pathogens (Rajapaksha et al., 2019). In the last decade, metagenomic next-generation sequencing (mNGS) technology has been applied for diagnosing infectious diseases through detection of microbial nucleic acids, but it is associated with complicated and uncertain results (Cummings et al., 2016; Simner et al., 2018). Thus, an ideal diagnostic method that can determine pathogens and guide antibiotic treatment rapidly before multiple organ dysfunction syndrome (MODS) develops is urgently needed.

Cell-free DNA (cfDNA) in plasma was first discovered in the last century. Circulating cfDNA molecules originate from dying cells and colonizing or invasive microorganisms, which release nucleic acids into the blood as they breakdown (Lee et al., 2020). At present, plasma mcfDNA sequencing technology is still relatively new, and its diagnostic performance in various invasive infections remains unclear. Therefore, this study was performed to compare the diagnostic ability of plasma mcfDNA sequencing with that of blood culture for sepsis in ICU patients and to verify whether plasma mcfDNA sequencing is a promising and non-invasive tool for monitoring and predicting infectious diseases.

## Methods

### Scope of the Research

Patients admitted to the ICU with suspected bloodstream infection (BSI) in Shanghai East Hospital from January 2020 to June 2020 were enrolled in the prospective study. Those with incomplete medical records or without paired results of plasma mcfDNA sequencing and blood culture were excluded. Clinical data were collected from the electronic medical record system, including baseline demographic characteristics, clinical diagnosis and prognosis as well as the relevant laboratory test results.

The patients were divided into an infectious disease group (ID group) and a non-infectious disease group (NID group) based on the final disease diagnosis by ICU specialists. Furthermore, the infectious disease group was divided into two subgroups according to the definition of sepsis-3.0 (Rhodes et al., 2016): a sepsis group and a non-sepsis group. Blood samples were simultaneously subjected to regular blood culture (BacT/Alert Virtuo, BioMérieux, France) and plasma mcfDNA sequencing testing (The Beijing Genomics Institute, China). Other clinical microbiological assays were also performed, including smear, culture of samples taken from relevant infected sites, and β-D-glucan/galactomannan (BDG/GM) tests, among others. The study was approved by the ethics committee of Shanghai East Hospital (No.2018-099) and was conducted in accordance with the Declaration of Helsinki (as revised in 2013). Written informed consent was obtained from all patients or their close relatives. Their personal information is not disclosed publicly.

### Plasma McfDNA Sequencing and Analysis

#### Sample Processing and DNA Extraction

Three to five milliliters of blood were drawn from each enrolled patient into the blood collection tubes. The blood collection tubes were centrifuged at 3,000 rpm at 4°C for 10 min, and 300 µL of plasma was taken into a new sterile tube, which could be frozen at −80°C to prevent nucleic acid degradation. The nucleic acid templates were extracted using TIANamp Micro DNA Kit (DP316, TIANGEN BIOTECH, China).

#### Construction of DNA Libraries and Sequencing

End repair, adapter ligation and PCR amplification were performed on the extracted nucleic acid to prepare a DNA library. DNA libraries were quantified using the Qubit dsDNA HS Assay Kit (Thermo Fisher Scientific, United States) and pooled in equal mass. The qualified DNA library pool was circularized to form a single-stranded circular structure, and rolling circle amplification (RCA) was used to generate DNA nanoball (DNB). The prepared DNB was loaded on the chip, and metagenomic sequencing platform BGISEQ-50 was used for sequencing.

#### Bioinformatic Analysis

After the sequencing data was offline, low-quality sequencing data was removed to obtain high-quality data. Through Burrows-Wheeler Alignment, the sequences aligned to the human reference genome (hg19) in the high-quality data were removed. The remaining data was simultaneously aligned to four microbial genome databases (PMSEQ® commercial databases) including bacteria, fungi, viruses, and parasites after removing low-complexity reads. RefSeq contains 6,350 bacterial genomes or scaffolds, 1,064 fungi, 1,798 DNA viruses and 234 parasites. The existence of pathogens was determined according to the following rules:

1) Bacteria (excluding *mycobacteria*), fungi, parasites, *mycoplasma/chlamydia/rickettsia*: microorganisms identified in one sample were sorted according to Standard Genus-Stringently Mapped Reads Number (SDG_SMRN). The top five list was obtained for further analysis. There were no more than two species for each genus, which were sorted according to Standard Species-Stringently Mapped Reads Number (Latin SMRN). The number of strictly mapped reads was ≥3.
2) Viruses: microorganism identified in one sample were sorted according to Standard Species-Stringently Mapped Reads Number (Latin SMRN). The top five lists were obtained for further analysis, and the number of strictly mapped reads was ≥3.
3) *Mycobacteria*: When the number of strictly mapped reads was ≥1, *Mycobacterium tuberculosis* (MTB) was considered positive. The principle of nontuberculous mycobacteria (NTM) identification is the same as that described above for principle (1).
4) In addition to SMRN, parameters such as coverage, depth and the Shannon index were considered comprehensively.

### Statistical Analysis

Data analyses were performed using SPSS 22.0 software. Comparative analysis was conducted by the Pearson χ2 test, McNemar test, or Fisher exact test. *p* values <0.05 were considered significant, and all tests were 2-tailed.

## Results

### Characteristics of Patients and Samples

**FIGURE 1:**
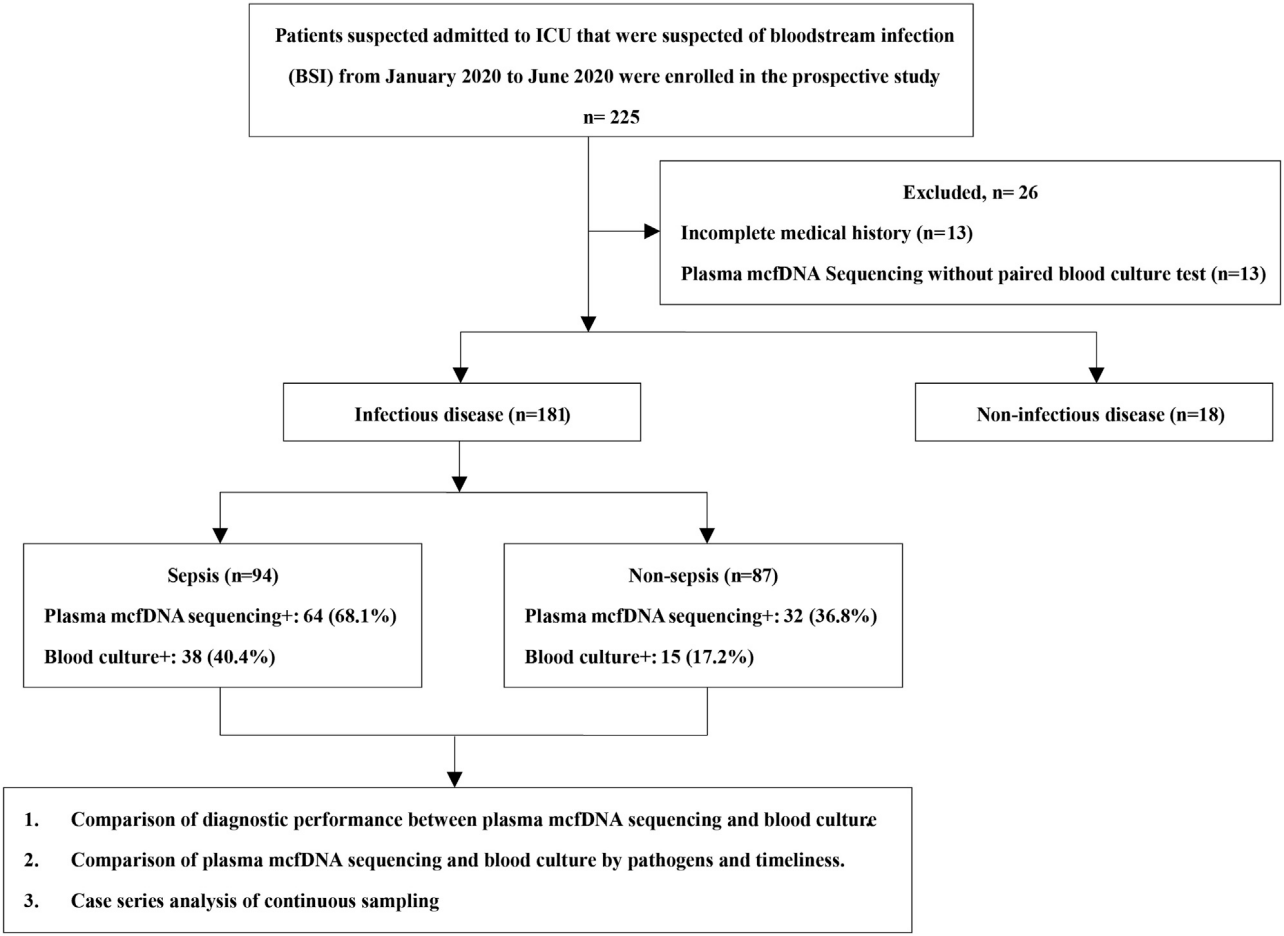
Flowchart of the Study. ICU, intensive care unit.

**FIGURE 2:**
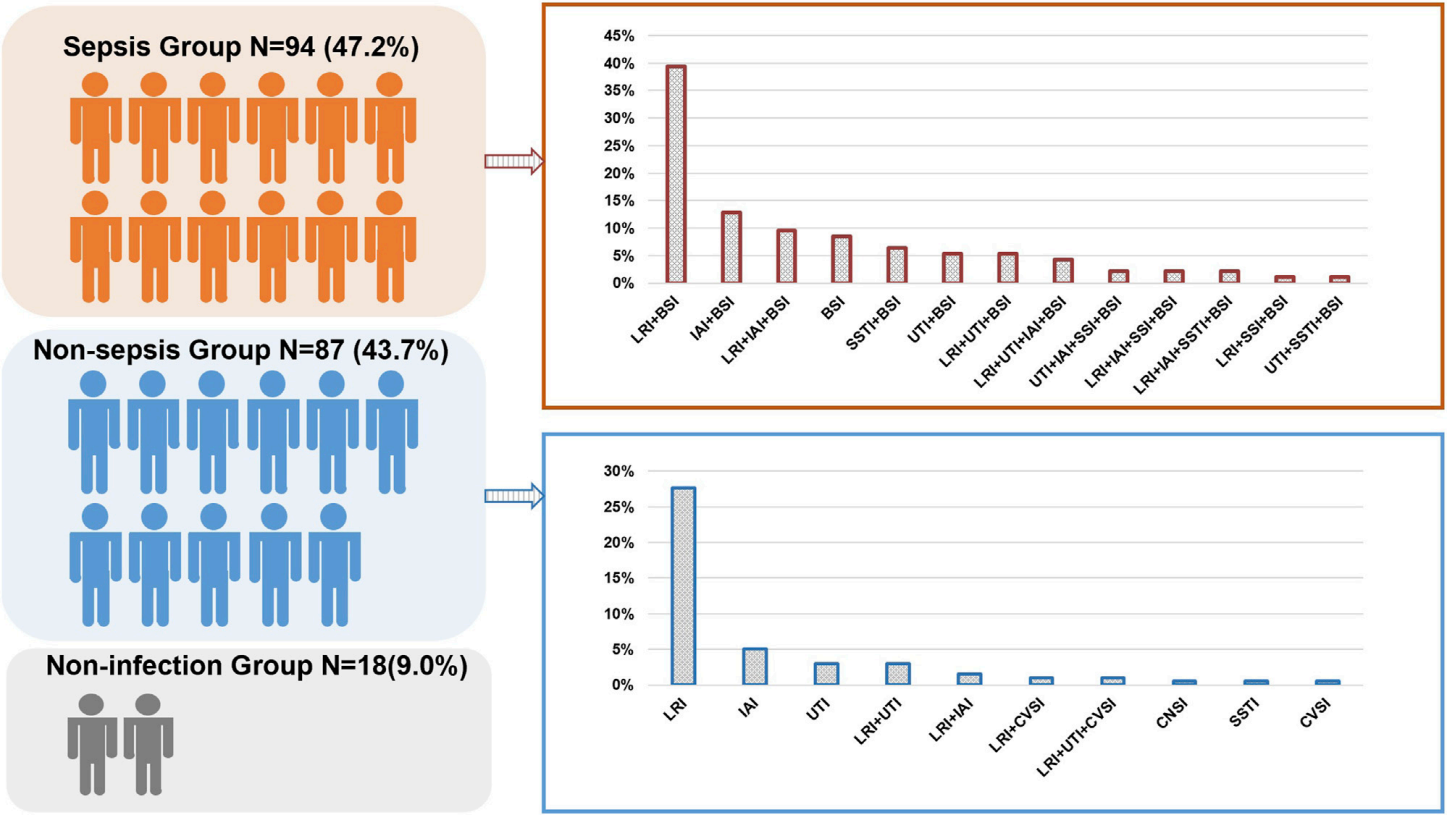
Distribution of the sepsis and non-sepsis group in the study. BSI, bloodstream infection; CVSI, cardiovascular infection; LRI, lower respiratory infection; SSTI, skin or soft-tissue infection; UTI, urinary tract infection; IAI, intra-abdominal infection; CNSI, central nervous system infection.

A total of 225 patients were screened, and 199 were enrolled in the study. Among them, 181 were diagnosed with an infectious disease (Figure 1). Among the patients, 64.8% were male. The average length of ICU stay was 13 days, and the 28-days mortality in-hospital was 27.6% (Supplementary Table S1). Repeat tests were performed on 102 occasions, involving 44 patients. In our study, BSI (94, 47.2%) was revealed as the most common infectious disease, followed by lower respiratory tract infections (LRTIs). In the non-sepsis group [87/181 (48.1%)], LRTIs were most common [68/87 (78.2%)], followed by urinary tract infections (UTIs) [14/87 (16.1%)] and intra-abdominal infections (IAIs) [13/87 (14.9%)] (Figure 2). In the sepsis group, 64 (68.1%) of samples were diagnosed with confirmed pathogens by both methods (Supplementary Table S2). Moreover, multiple consecutive microbial detections (sampling ≥3 times) were undertaken for 11 patients, eight of whom were diagnosed with sepsis.

### Performance of Plasma McfDNA Sequencing Relative to Blood Culture

#### Diagnostic Performance in Distinguishing Sepsis From Non-Sepsis

**FIGURE 3:**
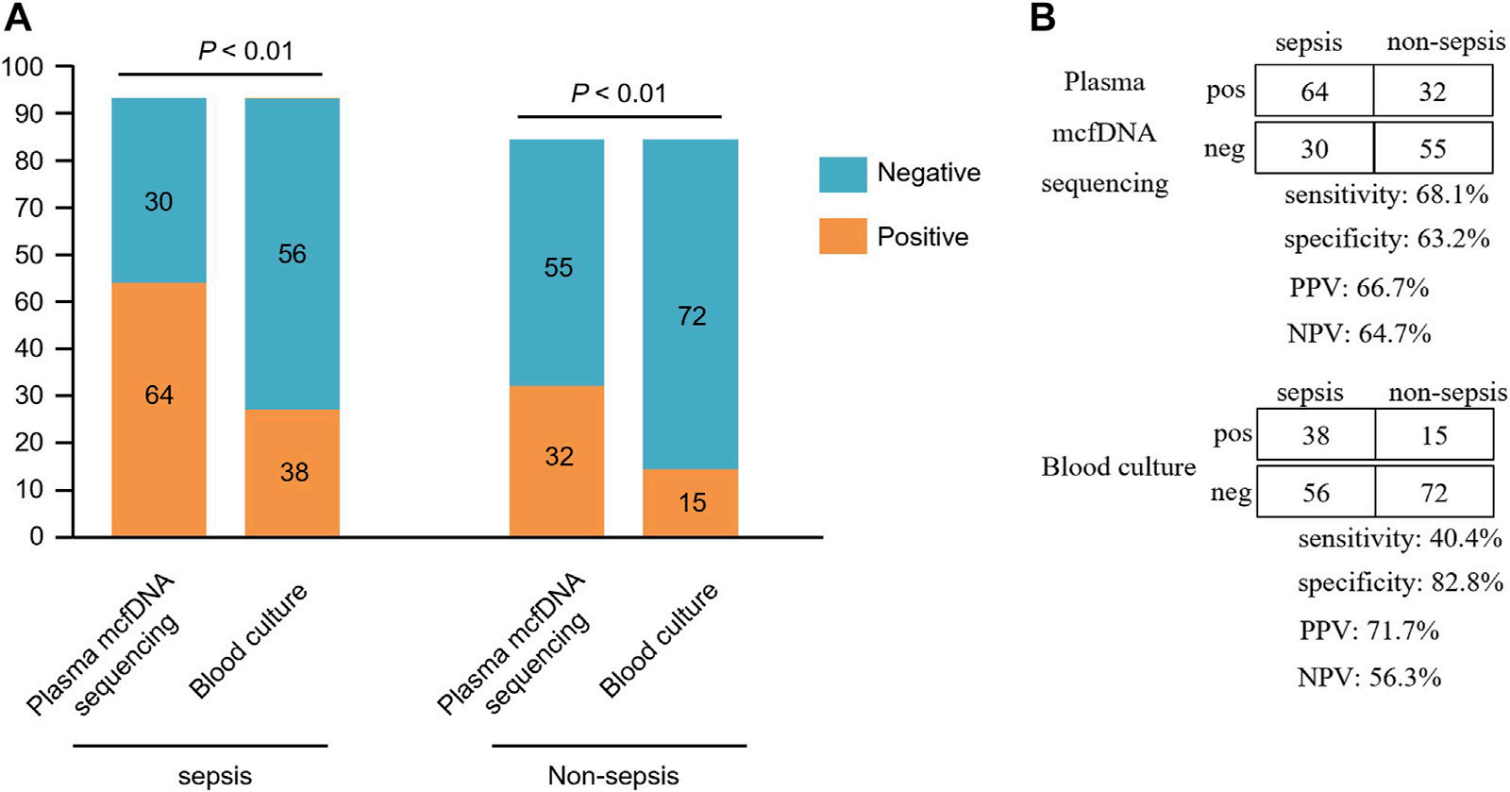
Comparison of plasma microbial cell-free DNA (mcfDNA) sequencing and blood culture for sepsis group and non-sepsis group. **(A)**, Number of positive results samples for pairwise plasma NGS and blood culture testing is plotted against the sepsis and non-sepsis. **(B)**, The diagnostic performance of plasma mcfDNA sequencing and blood culture testing for differentiating sepsis from non-sepsis is shown by the contingency tables. NPV, negative predictive value; PPV, positive predictive value. pos, positive; neg, negative.

In the sepsis group, 64 of the 94 mcfDNA sequencing tests had a positive result for at least one microorganism (Supplementary Table S2). Bacteria are the most commonly detected species, and 58 tests identified at least one bacterium. Excluding the NID group (*n* = 18), 181 patients were included in the diagnostic performance study. The positive rate for plasma mcfDNA sequencing was significantly higher than that for blood culture in both the sepsis and non-sepsis groups (*p* < 0.01) (Figure 3A). The positive predictive value (PPV), negative predictive value (NPV), positive likelihood ratio and negative likelihood ratio of diagnosing sepsis by plasma mcfDNA sequencing were 66.7%, 64.7%, 1.85, and 0.50, respectively. The sensitivity of plasma mcfDNA sequencing was significantly higher than that of blood culture (68.1 vs. 40.4%, *p* < 0.01), though plasma mcfDNA sequencing showed a decrease in specificity of approximately 20% compared with blood culture (63.2 vs. 82.8%, *p* < 0.01) (Figure 3B).

#### Correlation and Concordance Between Plasma McfDNA Sequencing and Blood Culture

**FIGURE 4:**
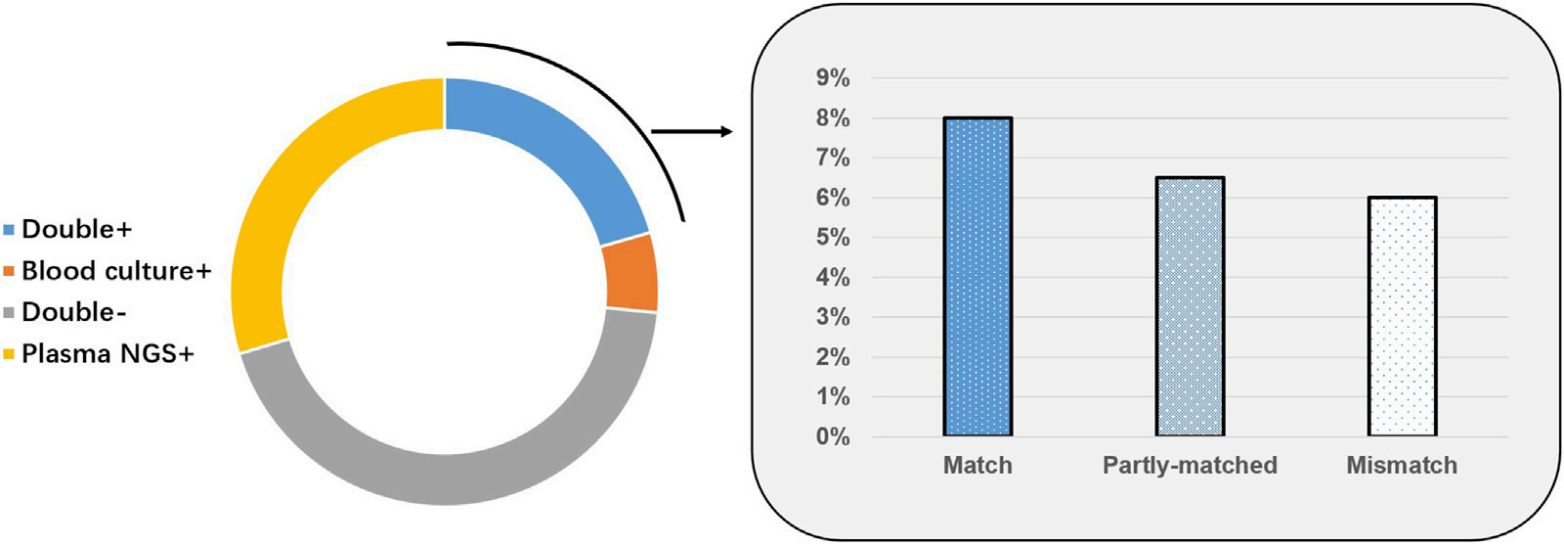
Correlation and concordance between plasma microbial cell-free DNA (mcfDNA) sequencing and blood culture for all samples (*n* = 199).

Of 199 cases, 41 (20.6%) were positive based on plasma mcfDNA sequencing and blood culture; 87 cases (43.7%) were negative. Fifty-nine (29.6%) cases were positive only by plasma mcfDNA sequencing, and 12 (6.0%) cases were positive only by blood culture. For the double-positive cases (*n* = 41), the results for 16 cases were matched completely, whereas the results for 12 cases were completely unmatched. The remaining 13 cases were “partially matched”; that is, at least one pathogen overlapped (Figure 4). In the non-sepsis group, the organisms identified by plasma mcfDNA sequencing were considered to be unlikely causes of sepsis, including a number of reactivated herpesviruses (4/29), chronic infections such as *Helicobacter pylori* (2/29), microorganisms likely to be commensals (9/29) and possible causes of non-sepsis-related acute infection (13/29) (Supplementary Table S3).

#### Effect of Antibiotic Exposure on Pathogen Detection

**FIGURE 5:**
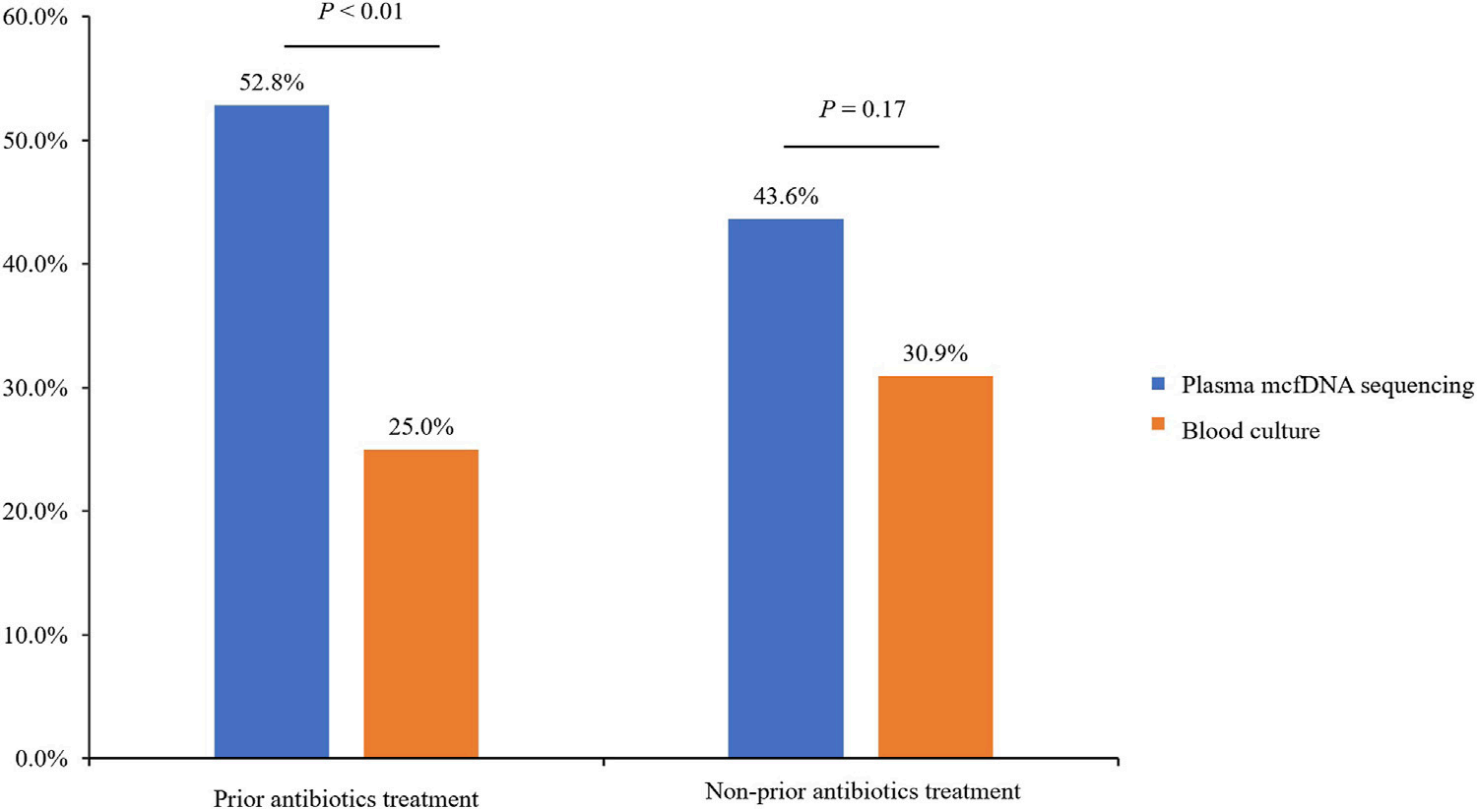
The effect of prior antibiotic exposure on plasma microbial cell-free DNA (mcfDNA) sequencing positivity.

All enrolled participants were admitted to the ICU; as most of them received endotracheal intubation for respiratory support after hospitalization, the exposure rate of antibiotics prior to plasma mcfDNA sequencing and blood culture was relatively high (144/199, 72.4%) in our analysis. Among patients not exposed to antibiotics (*n* = 55), there was no significant difference in the positive rate between plasma mcfDNA sequencing and blood culture (43.6 vs. 30.9%, *p* = 0.17). Due to the previous application of antibiotics (*n* = 144), the rate of plasma mcfDNA sequencing positivity was significantly higher than that of blood culture (52.8 vs. 25.0%, *p* < 0.01) (Figure 5).

### Comparison of Plasma McfDNA Sequencing and Blood Culture in Terms of Timeliness and Pathogens

#### Comparison Analysis in Terms of Timeliness

The eight most common pathogens of blood culture and their turnaround time (TAT) are listed in Table 1. The laboratory turnaround time from the initiation of blood sample processing by nucleic acid extraction to the completion of sequencing analysis (TAT3) was approximately 26 h using our platform. In contrast, the TAT of blood culture differed for various culturable pathogens. For example, the TAT1 (TAT from blood culture bottles to in and out of the Virtuo system) was the shortest for *Acinetobacter baumannii* detection, at only 9.4 h. Among eight pathogens, anaerobes required the longest time to detect (34.1 h). TAT1 was equivalent to that of plasma mcfDNA sequencing in terms of *Candida*. TAT2 (turnaround time from blood culture bottles to the Virtuo system to receive the reports) was significantly longer than TAT3 (*p* < 0.01).

**TABLE 1:** Comparison of plasma mcfDNA sequencing and blood culture testing in terms of timeliness.

Pathogens	TAT1^a^	P1^b^	TAT2^a^	P2^b^
*Acinetobacter baumannii*	9.4 (9.2–10.3)	<0.01	66.9 (49.0–77.8)	<0.01
*Klebsiella pneumoniae*	11.1 (10.2–14.4)	<0.01	66.3 (47.8–74.5)	<0.01
*Escherichia coli*	10.4 (7.9–13.2)	<0.01	66.5 (53.2–67.5)	<0.01
*Anaerobes*	34.1 (32.9–35.2)	0.33	87.2 (80.3–94.2)	<0.01
*Staphylococcus aureus*	15.9 (15.2–19.4)	<0.01	70.2 (50.2–87.2)	<0.01
*Enterococcus*	13.3 (12.1–15.1)	<0.01	64.2 (51.5–68.4)	<0.01
*Staphylococcus epidermidis/capitis/hominis/haemolyticus*	20.7 (17.7–29.7)	<0.01	73.2 (63.2–95.3)	<0.01
*Candida*	25.3 (18.6–28.5)	0.15	83.1 (73.1–92.8)	<0.01

a Data are presented as median (IQR) owing to non-normal distribution.

b Comparisons between the two groups were conducted using the Mann-Whitney *U* test. TAT1, the turnaround time from the blood culture bottles on the Virtuo to the off Virtuo; TAT2, the turnaround time from the blood culture bottles on the Virtuo to send reports; TAT3, the laboratory turnaround time from initiation of blood sample processing by nucleic acid extraction to completion of sequencing analysis was about 26 h by our platform.P1, comparison between the TAT1 and TAT3; P2, comparison between the TAT2 and TAT3. IQR, interquartile range. TAT, turnaround time.

#### Comparison Analysis in Terms of Pathogens

**FIGURE 6:**
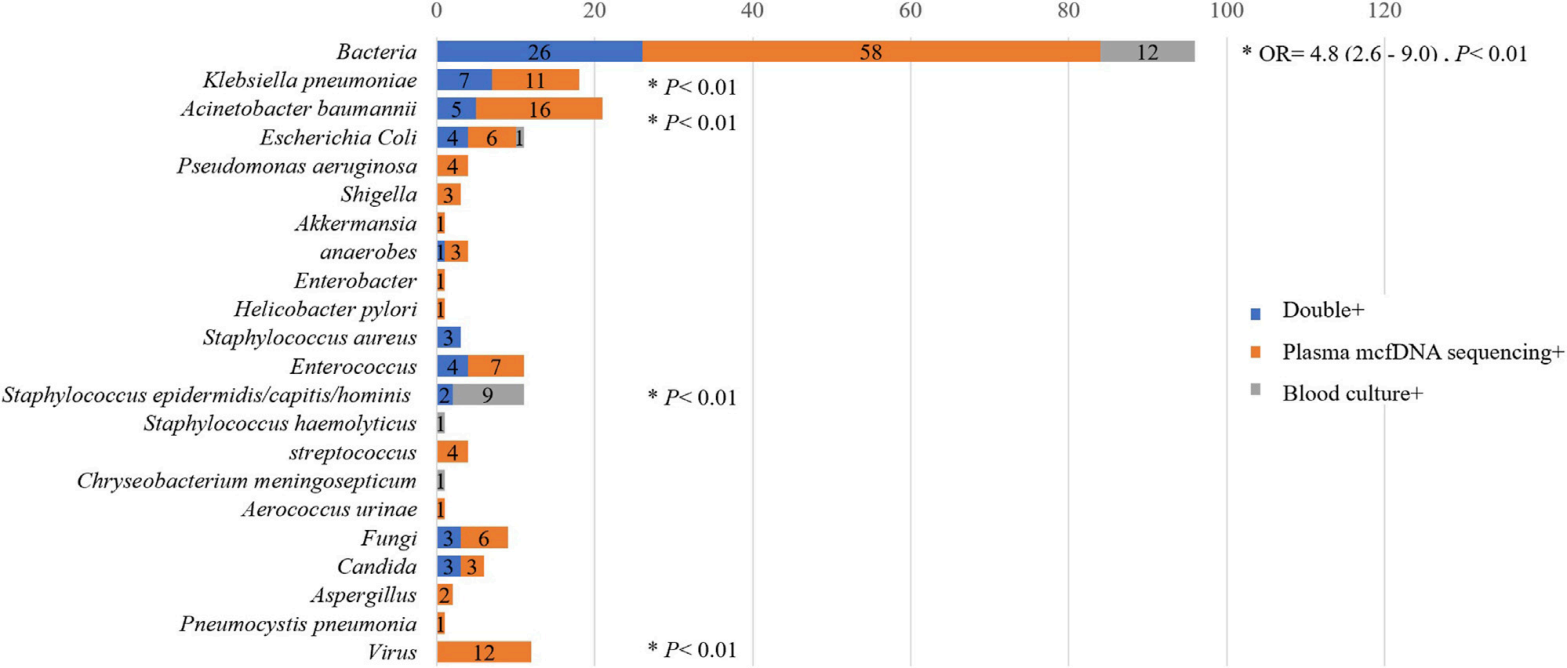
Comparison of plasma microbial cell-free DNA (mcfDNA) sequencing and blood culture testing by in terms of pathogens.

As shown in Figure 6, the microorganisms most frequently detected by both methods included *Acinetobacter baumannii, Klebsiella pneumoniae* and *Escherichia coli*. Overall, the percentage of plasma mcfDNA sequencing-positive samples was significantly higher than that of blood culture-positive samples in terms of bacteria {odds ratio [OR]= 4.8, [95% confidence interval (CI)= 2.6–9.0], *p* < 0.01}, *Acinetobacter baumannii* (*p* < 0.01)*, Klebsiella pneumoniae* (*p* < 0.01), *Enterococcus* (*p* = 0.02), fungi (*p* = 0.03) and viruses (plasma mcfDNA sequencing only, *p* < 0.01). Anaerobes were also observed to have a higher yield rate by plasma mcfDNA sequencing than by blood culture, though the difference was not significant due to the small sample size (*p* = 0.25).

#### Case Series Analysis of Continuous Sampling

Eleven patients were clinically considered to be in a state of complex infection, as described in Supplementary Table S4, and plasma mcfDNA sequencing tests were dynamically performed to determine the variation in microbiological species present in these patients during treatment. In the early stage of PT001′s severe pneumonia, *K. pneumoniae* was detected rapidly and accurately in blood samples by plasma mcfDNA sequencing, which showed good consistency with results of BALF samples by culture. With disease progression, an increase in *K. pneumoniae* burden in the blood was found, and coinfection by *K. pneumoniae*, *P. aeruginosa* and *A. baumannii* was revealed by plasma mcfDNA sequencing, resulting in aggravation to severe pneumonia and further sepsis development. PT043 was admitted to the ICU for respiratory failure secondary to nasopharyngeal carcinoma, and EBV nucleic acids in the blood cleared gradually. However, chemotherapy led to further immune dysfunction in patients, which reduced self-defense and aggravated *A. baumannii* infection.

## Discussion

Metagenomic next-generation sequencing has been widely used for pathogen detection in clinical practice (Wilson et al., 2019). The greatest benefit is that plasma mcfDNA sequencing is able to detect pathogens that are difficult to culture, thereby overcoming the shortcomings of traditional microbial methods. As a biopsy method for non-invasive disease diagnosis, it can diagnose possible infections by capturing and identifying highly fragmented mcfDNA in the blood. As 72.4% of the ICU patients in our study were treated with antibiotics before sampling, the detection rate of plasma mcfDNA sequencing was significantly higher than that of blood culture for the common bacteria that cause hospital-acquired infection, such as *Klebsiella pneumoniae* and *Acinetobacter baumannii*. The rate of *Staphylococcus epidermidis/capitis/hominis/haemolyticus* positivity based on plasma mcfDNA sequencing was significantly lower than that of blood culture (*p* < 0.01), which may be because the SDG_SMRN of *Staphylococcus* is not in the list of top five organisms.

In our study, potential pathogens in ICU patients were detected by conventional blood culture and plasma mcfDNA sequencing at the same time, and the latter had a high sensitivity (68.1%), approximately 28% higher than that of traditional blood culture, for detecting sepsis. This was partially attributable to technical shortfalls in blood culture acquisition as well as to local foci, fastidious organisms, or very low rates of viable microorganisms in the bloodstream. Patients with sepsis exhibit a significantly higher amount of cfDNA than healthy people because of the direct release of DNA from bacteria or co-release from phagocytic cells, which is thus detected by mNGS (Grumaz et al., 2016).

Plasma mcfDNA sequencing displayed lower specificity than that of blood culture (64.0 vs. 82.6%), with false positives likely causing by impaired mucosal barrier or immunosuppression, especially in cancer patients and transplant recipients (Han et al., 2020). In many cases, an invasive sampling technique is unbearable for ICU patients with conditions complicated by a variety of serious bacterial, fungal or viral infections. A single-centre study reported that 61% of plasma mcfDNA sequencing tests were positive for clinically relevant pathogens among immunocompromised hosts and a total of 34 invasive techniques were avoided due to mNGS results (Rossoff et al., 2019). Even if the infection is restricted to specific anatomical locations, plasma mcfDNA sequencing tests can detect pathogens’ nucleic acids (O'Grady, 2019). In our study, the 44.8% of pathogens (13/29) detected only by plasma mcfDNA sequencing in the non-sepsis group indicated infection in other parts of the body, such as LRI, IAI, and CNSI. The mcfDNA detected might be from an invasive pathogen opportunistically entering the bloodstream when the tissue mucosa was damaged, resulting in bacteraemia or viraemia in severe cases. Nevertheless, bacterial DNA is often present in the blood at low levels. According to the study of Gu et al., in matched samples, the load of pathogen cfDNA in body fluids was significantly higher than that in blood (Gu et al., 2021).

In addition to diagnosing infections from most bodily sources, plasma mcfDNA sequencing can detect a much wider pathogen spectrum than blood culture. More than two pathogens were detected in 32 (32/94, 34%) cases diagnosed with sepsis using plasma mcfDNA sequencing. In contrast, blood culture often only identifies a single species, which may be due to the competition of different types of microorganisms in the same environment (Wang et al., 2018). Plasma mcfDNA sequencing has also been used to detect viruses. There is still no uniform standard to determine whether the virus detected by plasma mcfDNA sequencing causes infection. In fact, this needs to be determined based on the patient’s medical history. For example, the human parvovirus B19 nucleic acid sequence was detected in the blood of PT132 (Supplementary Table S5). The patient’s clinical manifestations were cardiovascular symptoms (arrhythmia, heart failure, cardiogenic shock), consistent with the diagnosis of cardiomyopathy. B19 exhibits myocardial tropism and is considered a relatively common cause of myocarditis (Pollack et al., 2015). However, a multi-centre retrospective cohort study reported cases with positive Karius results, and the clinical impact did not involve DNA viruses or parasites but only bacteria and/or fungi (Hogan et al., 2021).

In China, lower respiratory infection is one of the most serious public health problems. A multi-centre clinical survey in China showed that the average incidence of hospital-acquired pneumonia (HAP) in the respiratory ward and respiratory ICU is 0.9 and 15.3%, respectively (Xie et al., 2020). Of note, our data indicated a high detection frequency of complications resulting from bloodstream infection in patients with severe pneumonia, warranting attention. A great deal of evidence has recently indicated that bloodstream infection is an important predictor of worsening prognosis in patients with severe pneumonia (Zhou et al., 2019), and early identification of sepsis or bloodstream infection in patients with severe pneumonia would help guide precise antimicrobial treatment. Moreover, Chen et al. found that in addition to metagenomic DNA, RNA-seq was able to identify a wide range of LRI pathogens, with improved sensitivity for viruses and fungi (Chen et al., 2020).

Limitations of our study include the following. Since there is no uniform reference standard for plasma mcfDNA sequencing, the gold standard for the diagnosis of infectious diseases was based on comprehensive evaluation of the infection group in this study, which included the interpretation of all microbial data, relevant clinical biomarkers and clinical adjudication. Confirmatory tests, such as bacterial 16S rDNA PCR or fungal 28 SrDNA-ITS PCR, were not performed on the samples. This study mainly evaluated the diagnostic performance of blood culture and plasma mcfDNA sequencing. As blood culture is not suitable for detecting viruses, RNA testing was not included in this study. We did not test samples other than blood samples by mcfDNA sequencing, and therefore some focal infections might have been missed, which would not affect the diagnosis of sepsis.

## Data Availability

All datasets in this study are included in the article/Supplementary Material and accompanying information is available in Sequence Read Archive database under accession number PRJNA705546.
