# Supplementary material for: Plasma Microbial Cell-Free DNA Sequencing Technology for the Diagnosis of Sepsis in the ICU
Source: Front Mol Biosci. 2021 May 28;8:659390. doi: 10.3389/fmolb.2021.659390 (PMC8194294; doi:10.3389/fmolb.2021.659390)
Supplement: Supplementary file 1 [file DataSheet1.docx]

**Supplemental Digital Content for** **Plasma Microbial Cell-Free DNA Sequencing Technology for the Diagnosis of Sepsis in ICU**

**Table of Contents**

**Table S1. Demographic Characteristic of the Patients Enrolled in the Study……2**

**Table S2. Pathogens Identified by Plasma Microbial Cell-Free DNA Sequencing Technology and Culture in the study ………………………………………………..3**

**Table S3. Pathogens Detected Only by Plasma NGS in the Non-sepsis Group…………………………………………………….. ……………….. ….. …..27**

**Table S4. Case Series Analysis of Continuous Sampling (≥3)…………………….28**

**Table S5. Viruses Detected by Plasma Microbial Cell-Free DNA Sequencing Technology…………………………………………………………………...………31**

| **Table S1. Demographic Characteristic of the Patients Enrolled in the Study (n=199)** | |
| --- | --- |
| **Variable** | **Value** |
| Age, median (range), years | 81 (25-98) |
| Sex, n (%) |  |
| Male | 129 (64.8) |
| Female | 70 (35.2) |
| 28day mortality in-hospital, n (%) | 55 (27.6) |
| Length of stay |  |
| Length of total hospital stay, median (IQR), d | 23 (13-36) |
| Length of ICU stay, median (IQR), d | 13 (6-24) |
| Infection sites, n (%) |  |
| Non-infection | 18 (9.0) |
| Single site infection | 82 (41.2) |
| Multiple sites infection | 99 (49.7) |
| Sampling numbers for one patient, n (%) |  |
| Once | 97 (68.8) |
| Twice | 33 (23.4) |
| Three times | 8 (5.7) |
| Four times | 3 (2.1) |

|  | | **Table S2. Pathogens Identified by Plasma Microbial Cell-Free DNA Sequencing Technology and Culture in the study (n=199)** | | | | | | | | | | | | | | |
| --- | --- | --- | --- | --- | --- | --- | --- | --- | --- | --- | --- | --- | --- | --- | --- | --- |
| **Patient No.** | **Sample No.** | **proved pathogen** | **Plasma NGS and blood culture** | | | | **Other microbiologic tests** | | | | | | | | | |
|  |  |  | **mcfDNA sequencing (Pathogens, No. of reads)** | **Blood culture** | **concordance** | **match** | **Sputum/BALF culture** | **Urine culture** | **CSF culture** | **Bile culture** | **Pleural/ ascitic fluid** | **Pus culture** | **rectal swab culture** | **Catheter culture** | **Secretion culture** | **Other** |
| PT001 | N20200001 | *Klebsiella pneumoniae, Acinetobacter baumannii* | *Klebsiella pneumoniae* 6 | *Staphylococcus capitis* | double-positive | mismatch | *Klebsiella pneumoniae, Acinetobacter baumannii* | */* | */* | */* | */* | */* | */* | */* | */* |  |
| PT001 | N20200009 | *Klebsiella pneumoniae, Acinetobacter baumannii* | *Klebsiella pneumoniae* 20*, Enterococcus faecium* 18 | *neg* | mNGS-positive | / | *Klebsiella pneumoniae, Acinetobacter baumannii* | */* | */* | */* | */* | */* | */* | */* | */* |  |
| PT001 | N20200014 | *Klebsiella pneumoniae, Acinetobacter baumannii* | *Klebsiella pneumoniae* 334*, Pseudomonas aeruginosa* 32, *Acinetobacter baumannii* 20 | *Klebsiella pneumoniae* | double-positive | match | *Klebsiella pneumoniae, Acinetobacter baumannii* | */* | */* | */* | */* | */* | */* | */* | */* |  |
| PT002 | N20200002 | */* | *neg* | *neg* | double-negative | / | */* | */* | */* | */* | */* | */* | */* | */* | */* |  |
| PT002 | N20200005 | *Acinetobacter baumannii* | *Acinetobacter baumannii* 3553 | *Acinetobacter baumannii* | double-positive | match | *Acinetobacter baumannii* | *Enterococcus faecium* | */* | */* | */* | */* | */* | */* | */* |  |
| PT003 | N20200003 | */* | *neg* | *neg* | double-negative | / | *Klebsiella pneumoniae* | *Enterococcus faecium, Candida glabrata* | */* | */* | */* | */* | */* | */* | */* |  |
| PT004 | N20200004 | */* | *neg* | *neg* | double-negative | / | */* | */* | */* | */* | */* | */* | */* | */* | */* |  |
| PT005 | N20200006 | *Escherichia Coli* | *Escherichia Coli* 273, *Shigella dysenteriae* 20 | *Escherichia Coli* | double-positive | partly-matched | *Candida glabrata* | */* | */* | *Escherichia Coli* | */* | */* | */* | */* | */* |  |
| PT005 | N20200012 | *Acinetobacter baumannii* | *Acinetobacter baumannii* 2472, *Escherichia Coli* 304, *Pseudomonas aeruginosa* 15 | *neg* | mNGS-positive | / | *Acinetobacter baumannii* | */* | */* | *Acinetobacter baumannii, Enterococcus faecium* | */* | */* | *Acinetobacter baumannii* | */* | */* |  |
| PT007 | N20200008 | *Streptococcus pneumoniae* | *Streptococcus pneumoniae* 19 | *Staphylococcus epidermidis,Staphylococcus capitis* | double-positive | mismatch | */* | *Acinetobacter baumannii* | */* | */* | */* | */* | */* | */* | */* | Urinary Streptococcus pneumoniae Ag: pos |
| PT007 | N20200017 | */* | *Pseudomonas aeruginosa* 12 | *neg* | mNGS-positive | / | *Enterobacter cloacae* | */* | */* | */* | */* | */* | */* | */* | */* |  |
| PT008 | N20200010 | */* | *neg* | *neg* | double-negative | / | */* | *Candida albican* | */* | */* | */* | */* | */* | */* | */* |  |
| PT008 | N20200013 | */* | *neg* | *neg* | double-negative | / | */* | *Candida albican* | */* | */* | */* | */* | */* | */* | */* |  |
| PT009 | N20200015 | */* | *Klebsiella pneumoniae* 44 | *neg* | mNGS-positive | / | *Klebsiella pneumoniae* | */* | */* | */* | */* | */* | */* | */* | */* |  |
| PT010 | N20200016 | */* | *neg* | *neg* | double-negative | / | *Klebsiella pneumoniae* | */* | */* | */* | */* | */* | */* | */* | */* |  |
| PT011 | N20200019 | */* | *neg* | *neg* | double-negative | / | *Candida albican* | */* | */* | */* | */* | */* | */* | */* | */* |  |
| PT011 | N20200022 | *Candida albican* | *neg* | *Escherichia Coli, Staphylococcus hominis, Chryseobacterium meningosepticum* | culture-positive | / | *Candida albican* | */* | */* | */* | */* | */* | */* | */* | */* |  |
| PT011 | N20200053 | *Acinetobacter baumannii* | *Acinetobacter baumannii* 273, *Enterococcus faecium* 14 | *neg* | mNGS-positive | / | *Acinetobacter baumannii* | *Candida albican* | */* | */* | *Candida parapsilosis* | */* | */* | */* | */* |  |
| PT012 | N20200020 | */* | *neg* | *neg* | double-negative | / | */* | *Klebsiella pneumoniae* | */* | */* | */* | */* | */* | */* | */* |  |
| PT013 | N20200021 | *Enterococcus faecium,Enterococcus faecalis，Escherichia Coli* | *Enterococcus faecium*120, *Enterococcus faecalis*55, *Escherichia Coli* 26 | *Enterococcus faecium, Enterococcus faecalis* | double-positive | partly-matched | */* | */* | */* | */* | */* | */* | */* | */* | */* |  |
| PT014 | N20200023 | *Klebsiella pneumoniae* | *Klebsiella pneumoniae* 15 | *Klebsiella pneumoniae* | double-positive | match | */* | */* | */* | */* | *Klebsiella pneumoniae* | */* | */* | */* | */* |  |
| PT015 | N20200025 | */* | *neg* | *neg* | double-negative | / | */* | */* | */* | */* | */* | */* | */* | */* | */* |  |
| PT016 | N20200026 | */* | *neg* | *neg* | double-negative | / | */* | */* | */* | */* | */* | */* | */* | */* | */* |  |
| PT017 | N20200027 | *Enterococcus faecium,Aspergillus fumigatus* | *Enterococcus faecium* 392, *Aspergillus fumigatus* 49 | *neg* | mNGS-positive | / | */* | */* | */* | *Enterococcus faecium* | */* | *Enterococcus faecium, Aspergillus* |  | */* | */* |  |
| PT017 | N20200046 | *Acinetobacter baumannii,Enterococcus faecium,Candida parapsilosis* | *Acinetobacter baumannii* 556, *Enterococcus faecium* 44, *Enterobacter hormaechei* 31, *Candida parapsilosis* 36, *CMV* 1514 | *Candida parapsilosis* | double-positive | partly-matched | *Acinetobacter baumannii* | *Candida parapsilosis* | */* | *Acinetobacter baumannii* |  | *Acinetobacter baumannii, Aspergillus* | */* | *Candida parapsilosis* | */* |  |
| PT018 | N20200028 | */* | *neg* | *neg* | double-negative | / | *Acinetobacter baumannii* | */* | */* | */* | */* | */* | */* | */* | */* |  |
| PT018 | N20200031 | */* | *neg* | *neg* | double-negative | / | *Acinetobacter baumannii* | */* | */* | */* | */* | */* | */* | */* | */* |  |
| PT019 | N20200029 | */* | *Staphylococcus epidermidis* 7 | *Staphylococcus epidermidis* | double-positive | match |  | */* | */* | */* | */* | */* | */* | */* | */* |  |
| PT020 | N20200030 | */* | *neg* | *neg* | double-negative | / | *Candida albican* | */* | */* | */* | */* | */* | */* | */* | */* |  |
| PT022 | N20200033 | */* | *Klebsiella pneumoniae*16 | *neg* | mNGS-positive | / | */* | */* | */* | */* | */* | */* | */* | */* | */* |  |
| PT023 | N20200034 | */* | *neg* | *neg* | double-negative | / | */* | */* | */* | */* | */* | */* | *Acinetobacter baumannii* | */* | */* |  |
| PT023 | N20200038 | */* | *Acinetobacter baumannii* 10 | *Staphylococcus hominis* | double-positive | mismatch | */* | */* | */* | */* | */* | */* | *Acinetobacter baumannii* | */* | */* |  |
| PT024 | N20200035 | *Candida albican* | *Candida albicans* 14 | *Staphylococcus capitis* | double-positive | mismatch | *Candida albican* | */* | */* | */* | */* | */* | */* | */* | */* |  |
| PT025 | N20200036 | */* | *neg* | *neg* | double-negative | / | *Candida albican* | *Candida albican* | */* | */* | */* | */* | */* | */* | */* |  |
| PT026 | N20200037 | */* | *neg* | *neg* | double-negative | / | */* | */* | */* | */* | */* | */* | */* | */* | */* |  |
| PT026 | N20200062 | *Acinetobacter baumannii* | *Acinetobacter baumannii* 12, *CMV* 154, *HSV-1* 11 | *neg* | mNGS-positive | / | *Acinetobacter baumannii* | */* | */* | */* | */* | */* | */* | */* | */* |  |
| PT027 | N20200039 | *Escherichia Coli* | *Staphylococcus capitis* 6 | *Staphylococcus capitis* | double-positive | match | *Escherichia Coli* | */* | */* | *Escherichia Coli* | */* | */* | */* | */* | */* |  |
| PT028 | N20200041 | *Klebsiella pneumoniae, Acinetobacter baumannii* | *Klebsiella pneumoniae*237, *Acinetobacter baumannii* 264, *CMV* 32*，TTV* 21 | *neg* | mNGS-positive | / | *Klebsiella pneumoniae, Acinetobacter baumannii* | */* | */* | */* | */* | */* | */* | */* | */* |  |
| PT029 | N20200043 | */* | *Streptococcus salivarius* 87, *Streptococcus parasanguinis* 81 | *neg* | mNGS-positive | / | */* | */* | */* | */* | */* | */* | */* | */* | */* |  |
| PT030 | N20200044 | */* | *neg* | *neg* | double-negative | / | */* | *Candida albican* | */* | */* | */* | */* | */* | */* | */* |  |
| PT030 | N20200048 | *Staphylococcus aureus* | *Staphylococcus aureus*65 | *Staphylococcus aureus* | double-positive | match | */* | *Candida albican* | */* | */* | */* | */* | */* | */* | */* |  |
| PT031 | N20200045 | *Enterococcus faecalis* | *Escherichia Coli* 5741, *Enterococcus faecalis*405, *Shigella boydii* 19, *Shigella dysenteriae*14 | *Enterococcus faecalis* | double-positive | mismatch | */* |  | */* | */* | */* | */* | */* | */* | */* |  |
| PT033 | N20200049 | */* | *neg* | *neg* | double-negative | / | */* | */* | */* | */* | */* | */* | */* | */* | */* |  |
| PT034 | N20200052 | */* | *neg* | *neg* | double-negative | / | *Klebsiella pneumoniae* | */* | */* | */* | */* | */* | */* | */* | */* |  |
| PT035 | N20200054 | *Escherichia Coli* | *Escherichia Coli* 438, *Shigella boydii* 20, *Shigella dysenteriae* 16, *Clostridium perfringens*32 | *Staphylococcus epidermidis* | double-positive | mismatch | */* | */* | */* | *Escherichia Coli, Enterococcus faecalis* | */* | */* | */* | */* | */* |  |
| PT035 | N20200056 | */* | *neg* | *neg* | double-negative | / | */* | */* | */* | *Candida albican* | */* | *Escherichia Coli, Candida albican* | */* | */* | */* |  |
| PT036 | N20200055 | */* | *neg* | *neg* | double-negative | / | */* | */* | */* | */* | */* | */* | */* | */* | */* |  |
| PT037 | N20200057 | *Pneumocystis jirovecii* | *Pneumocystis jirovecii* 291 | *neg* | mNGS-positive | / | */* | */* | */* | */* | */* | */* | */* | */* | */* | BDG：240.37pg/ml |
| PT038 | N20200058 | */* | *neg* | *neg* | double-negative | / | *Klebsiella pneumoniae* | */* | */* | */* | */* | */* | */* | */* | */* |  |
| PT039 | N20200059 | *Acinetobacter baumannii* | *Acinetobacter baumannii* 12 | *neg* | mNGS-positive | / | *Acinetobacter baumannii* | */* | */* | */* | */* | */* | *Klebsiella pneumoniae* | */* | */* |  |
| PT039 | N20200066 | */* | *neg* | *neg* | double-negative | / | *Acinetobacter baumannii* | */* | */* | */* | */* | */* | */* | */* | */* |  |
| PT039 | N20200075 | */* | *neg* | *neg* | double-negative | / | *Acinetobacter baumannii* | */* | */* | */* | */* | */* | */* | */* | */* |  |
| PT039 | N20200107 | */* | *neg* | *neg* | double-negative | / | *Klebsiella pneumoniae* | */* | */* | */* | */* | */* | */* | */* | */* |  |
| PT040 | N20200060 | */* | *neg* | *neg* | double-negative | / | */* | *Candida tropical, Enterococcus faecium* | */* | */* | */* | */* | */* | */* | */* |  |
| PT041 | N20200063 | */* | *neg* | *neg* | double-negative | / | *Candida glabrata* | *Candida tropical* | */* | */* | */* | */* | */* | */* | */* |  |
| PT043 | N20200065 | */* | *EBV* 104 | *neg* | mNGS-positive | / | */* | */* | */* | */* | */* | */* | */* | */* | */* |  |
| PT043 | N20200074 | *Acinetobacter baumannii,Candida albican* | *Acinetobacter baumannii* 111, *EBV* 13 | *neg* | mNGS-positive | / | *Acinetobacter baumannii, Candida albican* | */* | */* | */* | */* | */* | */* | */* | */* |  |
| PT043 | N20200083 | *Acinetobacter baumannii* | *Acinetobacter baumannii* 1689 | *neg* | mNGS-positive | / | */* | */* | */* | */* | */* | */* | */* | */* | */* |  |
| PT044 | N20200067 | *Klebsiella pneumoniae* | *Klebsiella pneumoniae* 2898 | *Klebsiella pneumoniae* | double-positive | match | */* | */* | */* | */* | */* | *Klebsiella pneumoniae* | */* | */* | */* |  |
| PT044 | N20200069 | *Klebsiella pneumoniae* | *Klebsiella pneumoniae* 7138 | *neg* | mNGS-positive | / | *Acinetobacter baumannii* | */* | */* | */* | */* | *Klebsiella pneumoniae* | */* | */* | */* |  |
| PT045 | N20200117 | */* | *neg* | *neg* | double-negative | / | */* | */* | */* | */* | */* | */* | */* | */* | */* |  |
| PT046 | N20200068 | *Klebsiella pneumoniae* | *Klebsiella pneumoniae* 293 | *Klebsiella pneumoniae* | double-positive | match | */* | */* | */* | */* | */* | */* | *Klebsiella pneumoniae* | */* | *Proteus mirabilis* |  |
| PT047 | N20200070 | */* | *neg* | *neg* | double-negative | / | */* | */* | */* | */* | */* | */* | */* | */* | */* |  |
| PT048 | N20200071 | */* | *Staphylococcus epidermidis* 26 | *Staphylococcus capitis, Staphylococcus epidermidis* | double-positive | partly-matched | *Staphylococcus aureus, Acinetobacter baumannii* | */* | */* | */* | */* | */* | */* | */* | */* |  |
| PT049 | N20200072 | *Staphylococcus aureus 1625* | *Staphylococcus aureus* 1625, *CMV* 11 | *Staphylococcus aureus* | double-positive | partly-matched | */* | */* | */* | */* | */* | */* | */* | */* | */* |  |
| PT050 | N20200073 | *Klebsiella pneumoniae* | *Klebsiella pneumoniae* 5 | *neg* | mNGS-positive | / | *Klebsiella pneumoniae* | */* | */* | */* | */* | */* | */* | */* | */* | Endotoxin：175.06pg/ml |
| PT051 | N20200076 | */* | *neg* | *neg* | double-negative | / | */* | */* | */* | */* | */* | */* | */* | */* | */* |  |
| PT052 | N20200077 | */* | *neg* | *Staphylococcus epidermidis* | culture-positive | / | */* | */* | */* | */* | */* | */* | */* | */* | */* |  |
| PT052 | N20200090 | */* | *Pseudomonas aeruginosa* 19 | *neg* | mNGS-positive | / | */* | */* | */* | */* | */* | */* | */* | */* | */* |  |
| PT053 | N20200079 | */* | *neg* | *neg* | double-negative | / | *Staphylococcus aureus* | */* | */* | */* | */* | */* | */* | */* | */* |  |
| PT054 | N20200080 | */* | *neg* | *neg* | double-negative | / | */* | */* | */* | */* | */* | */* | */* | */* | */* |  |
| PT055 | N20200081 | *Escherichia Coli* | *Escherichia coli* 4 | *Escherichia coli* | double-positive | match | */* | *Escherichia Coli* | */* | */* | */* | */* | */* | */* | */* |  |
| PT055 | N20200088 | *Escherichia Coli* | *Klebsiella pneumoniae*72, *Escherichia coli*16 | *neg* | mNGS-positive | / | */* | *Escherichia Coli* | */* | */* | */* | */* | */* | */* | */* |  |
| PT056 | N20200082 | *Escherichia Coli* | *Escherichia coli* 34 | *neg* | mNGS-positive | / | */* | *Escherichia Coli* | */* | */* | */* | */* | */* | */* | */* |  |
| PT057 | N20200084 | */* | *neg* | *neg* | double-negative | / | */* | */* | */* | */* | */* | */* | */* | */* | */* |  |
| PT058 | N20200085 | */* | *HBV* 608 | *neg* | mNGS-positive | / | */* | */* | */* | */* | */* | */* | */* | */* | */* | HBsAg+,HBeAg+,HBcAb+ |
| PT059 | N20200086 | */* | *Klebsiella pneumoniae* 33 | *neg* | mNGS-positive | / | *Klebsiella pneumoniae* | */* | */* | */* | */* | */* | */* | */* | */* |  |
| PT060 | N20200087 | */* | *Staphylococcus capitis* 4 | *Staphylococcus capitis* | double-positive | match | */* | */* | */* | */* | */* | */* | */* | */* | */* |  |
| PT061 | N20200089 | */* | *neg* | *neg* | double-negative | / | */* | */* | */* | */* | */* | *Klebsiella acidogenes, Streptococcus agalactis* | */* | */* | *Klebsiella acidogenes, Streptococcus agalactis* |  |
| PT061 | N20200094 | */* | *neg* | *neg* | double-negative | / | */* | */* | */* | */* | */* | *Klebsiella acidogenes, Streptococcus agalactis* | */* | */* | *Klebsiella acidogenes, Streptococcus agalactis* |  |
| PT062 | N20200091 | *Klebsiella pneumoniae* | *Klebsiella pneumoniae* 79, *Helicobacter pylori* 26 | *Klebsiella pneumoniae* | double-positive | partly-matched | *Klebsiella pneumoniae* | */* | */* | */* | */* | */* | */* | */* | */* |  |
| PT064 | N20200093 | */* | *neg* | *neg* | double-negative | / | */* | */* | */* | */* | */* | */* | */* | */* | */* |  |
| PT064 | N20200099 | */* | *neg* | *neg* | double-negative | / | */* | */* | */* | */* | */* | */* | */* | */* | */* |  |
| PT065 | N20200095 | *Candida albican* | *Candida albican* 54 | *Candida albican* | double-positive | match | *Staphylococcus aureus, Enterobacter cloacae* | */* | */* | */* | */* | *Enterobacter cloacae* | */* | */* | */* |  |
| PT065 | N20200100 | *Candida albican* | *neg* | *Staphylococcus capitis* | culture-positive | / | *Klebsiella pneumoniae* | */* | */* | */* | */* | */* | */* | *Candida albican* | */* |  |
| PT066 | N20200096 | */* | *neg* | *neg* | double-negative | / | *Candida albican* | */* | */* | */* | */* | */* | */* | */* | */* |  |
| PT067 | N20200097 | *Enterococcus faecium* | *neg* | *Staphylococcus epidermidis* | culture-positive | / | */* | */* | */* | */* | */* | */* | */* | */* | */* |  |
| PT067 | N20200098 | *Enterococcus faecium, EBV* | *Enterococcus faecium* 28, *EBV* 1364 | *Enterococcus faecium* | double-positive | partly-matched | */* | */* | */* | */* | */* | */* | */* | */* | */* |  |
| PT068 | N20200101 | */* | *Pseudomonas aeruginosa* 15 | *neg* | mNGS-positive | / | */* | */* | */* | */* | */* | */* | */* | */* | */* | Endotoxin: 154.66 pg/mL |
| PT069 | N20200102 | */* | *CMV* 55 | *neg* | mNGS-positive | / | */* | */* | */* | */* | */* | */* | */* | */* | */* |  |
| PT070 | N20200103 | */* | *Tannerella forsythia* 787, *Prevotella intermedia* 90, *Prevotella denticola* 70, *Treponema socranskii* 77, *Treponema denticola* 76, *Streptococcus oralis* 25, *Streptococcus sanguinis* 24 | *neg* | mNGS-positive | / | */* | */* | */* | */* | */* | */* | */* | */* | */* |  |
| PT071 | N20200104 | */* | *neg* | *neg* | double-negative | / | */* | */* | */* | */* | *Candida albican* | */* | */* | */* | */* |  |
| PT072 | N20200105 | */* | *Serratia marcescens* 55, *Staphylococcus aureus*24, *Klebsiella pneumoniae* 16 | *neg* | mNGS-positive | / | *Staphylococcus aureus, Klebsiella pneumoniae* | */* | */* | */* | */* | */* | */* | */* | */* |  |
| PT072 | N20200110 | */* | *neg* | *neg* | double-negative | / | *Staphylococcus aureus, Klebsiella pneumoniae* | */* | */* | */* | */* | */* | */* | */* | */* |  |
| PT073 | N20200106 | */* | *neg* | *neg* | double-negative | / | */* | */* | */* | */* | */* | */* | */* | */* | */* |  |
| PT074 | N20200108 | *Candida albican* | *Candida albicans* 10 | *neg* | mNGS-positive | / | */* | */* | */* | */* | */* | */* | */* | */* | */* | BDG：67.86 pg/mL |
| PT075 | N20200109 | */* | *neg* | *neg* | double-negative | / | */* | */* | */* | */* | */* | */* | */* | */* | */* |  |
| PT075 | N20200119 | */* | *neg* | *neg* | double-negative | / | */* | */* | */* | */* | */* | */* | */* | */* | */* |  |
| PT076 | N20200111 | *Acinetobacter baumannii，Klebsiella pneumoniae* | *Acinetobacter baumannii* 870, *Klebsiella pneumoniae* 94 | *neg* | mNGS-positive | / | */* | *Candida albican* | */* | */* | */* | */* | */* | */* | */* | Endotoxin：206.46 pg/mL |
| PT077 | N20200112 | */* | *neg* | *neg* | double-negative | / | */* | */* | */* | */* | */* | */* | */* | */* | */* |  |
| PT078 | N20200113 | */* | *Pseudomonas aeruginosa* 21 | *neg* | mNGS-positive | / | */* | */* | */* | */* | */* | */* | */* | */* | */* |  |
| PT078 | N20200114 | */* | *neg* | *Staphylococcus haemolyticus* | culture-positive | / | */* | */* | */* | */* | */* | */* | */* | */* | */* |  |
| PT078 | N20200121 | */* | *neg* | *Staphylococcus haemolyticus* | culture-positive | / | */* | */* | */* | */* | */* | */* | */* | */* | */* |  |
| PT078 | N20200122 | */* | *neg* | *neg* | double-negative | / | */* | */* | */* | */* | */* | */* | */* | */* | */* |  |
| PT079 | N20200116 | *Enterococcus faecalis* | *Enterococcus faecalis* 1542, *Enterococcus faecium* 162 | *Enterococcus faecalis* | double-positive | partly-matched | */* | */* | */* | */* | */* | */* | */* | */* | */* |  |
| PT079 | N20200149 | *Enterococcus faecalis* | *Enterococcus faecium* 28, *Human parvovirus B19* 537 | *neg* | mNGS-positive | / | */* | */* | */* | */* | */* | *Enterococcus faecalis* | */* | */* | */* |  |
| PT081 | N20200123 | *Staphylococcus aureus* | *Acinetobacter baumannii* 14, *Staphylococcus aureus* 4, *Streptococcus parasanguinis* 9 | *Staphylococcus aureus* | double-positive | partly-matched | *Staphylococcus aureus, Klebsiella pneumoniae, Candida albican* | *Staphylococcus aureus* | */* | */* | */* | */* | */* | */* | */* |  |
| PT081 | N20200130 | *Klebsiella pneumoniae* | *Staphylococcus epidermidis* 4, *Klebsiella pneumoniae* 6 | *Staphylococcus epidermidis* | double-positive | mismatch | */* | */* | */* | */* | */* | */* | */* | */* | */* |  |
| PT083 | N20200125 | */* | *HSV-1* 1222 | *neg* | mNGS-positive | / | *Acinetobacter baumannii* | */* | */* | */* | */* | */* | */* | */* | */* |  |
| PT084 | N20200126 | */* | *neg* | *neg* | double-negative | / | */* | */* | */* | */* | */* | */* | */* | */* | */* |  |
| PT084 | N20200128 | */* | *neg* | *neg* | double-negative | / | */* | */* | */* | */* | */* | */* | */* | */* | */* |  |
| PT085 | N20200127 | */* | *neg* | *neg* | double-negative | / | */* | */* | */* | */* | */* | */* | */* | */* | */* |  |
| PT086 | N20200129 | */* | *Pseudomonas aeruginosa* 21 | *neg* | mNGS-positive | / | */* | */* | */* | */* | */* | */* | */* | */* | */* |  |
| PT087 | N20200131 | */* | *Helicobacter pylori* 88 | *neg* | mNGS-positive | / | *Aspergillus* | */* | */* | */* | */* | */* | */* | */* | */* |  |
| PT088 | N20200133 | */* | *Pseudomonas aeruginosa* 5 | *Streptococcus pneumoniae* | double-positive | mismatch | */* | */* | */* | */* | */* | */* | */* | */* | */* |  |
| PT088 | N20200142 | */* | *neg* | *Staphylococcus capitis* | culture-positive | / | */* | */* | */* | */* | */* | */* | */* | */* | */* |  |
| PT089 | N20200134 | */* | *neg* | *neg* | double-negative | / | */* | */* | */* | */* | */* | */* | */* | */* | */* |  |
| PT089 | N20200136 | */* | *neg* | *neg* | double-negative | / | */* | */* | */* | */* | */* | */* | */* | */* | */* |  |
| PT089 | N20200140 | */* | *neg* | *neg* | double-negative | / | *Citrobacter freundii* | */* | */* | */* | */* | */* | */* | */* | */* |  |
| PT090 | N20200135 | */* | *Helicobacter pylori* 70 | *neg* | mNGS-positive | / | */* | */* | */* | */* | */* | */* | */* | */* | */* |  |
| PT091 | N20200137 | */* | *neg* | *neg* | double-negative | / | *Streptococcus pneumoniae, Candida glabrata* | */* | */* | */* | */* | */* | */* | */* | */* |  |
| PT092 | N20200138 | */* | *neg* | *neg* | double-negative | / | */* | */* | */* | */* | */* | */* | */* | */* | */* |  |
| PT093 | N20200139 | */* | *neg* | *neg* | double-negative | / | *Klebsiella pneumoniae, Streptococcus pneumoniae* | */* | */* | */* | */* | */* | */* | */* | */* |  |
| PT095 | N20200143 | */* | *neg* | *neg* | double-negative | / | */* | */* | */* | */* | */* | */* | */* | */* | */* |  |
| PT097 | N20200145 | */* | *neg* | *neg* | double-negative | / | */* | */* | */* | */* | */* | */* | */* | */* | */* |  |
| PT097 | N20200148 | *Acinetobacter baumannii* | *Acinetobacter baumannii* 11 | *neg* | mNGS-positive | / | */* | */* | */* | */* | */* | */* | */* | */* | */* |  |
| PT097 | N20200151 | *Acinetobacter baumannii* | *Enterococcus faecium* 4, *Aspergillus fumigatus* 3, *HSV-1* 16 | *neg* | mNGS-positive | / | */* | */* | */* | */* | */* | */* | */* | */* | */* |  |
| PT098 | N20200146 | *Acinetobacter baumannii* | *Acinetobacter baumannii 18* | *neg* | mNGS-positive | / | *Candida albican* | *Candida albican* | */* | */* | */* | */* | */* | */* | */* |  |
| PT099 | N20200147 | */* | *neg* | *neg* | double-negative | / | */* | */* | */* | */* | */* | */* | */* | */* | */* |  |
| PT100 | N20200150 | */* | *neg* | *Staphylococcus hominis, Staphylococcus epidermidis, Staphylococcus aureus* | culture-positive | / | *Pseudomonas aeruginosa* | *Enterococcus faecalis、Candida glabrata* | */* | */* | */* | */* | */* | */* | */* |  |
| PT100 | N20200163 | */* | *neg* | *neg* | double-negative | / | */* | *Candida glabrata* | */* | */* | */* | */* | */* | */* | */* |  |
| PT100 | N20200165 | */* | *neg* | *neg* | double-negative | / | */* | */* | */* | */* | */* | */* | */* | */* | */* |  |
| PT101 | N20200152 | *Candida glabrata* | *Aerococcus urinae* 15 | *neg* | mNGS-positive | / | *Candida glabrata* | */* | */* | */* | */* | */* | */* | */* | */* |  |
| PT102 | N20200154 | */* | *neg* | *neg* | double-negative | / | */* | */* | */* | */* | */* | */* | */* | */* | */* |  |
| PT103 | N20200155 | */* | *neg* | *neg* | double-negative | / | */* | */* | */* | */* | */* | */* | */* | */* | */* |  |
| PT104 | N20200156 | */* | *neg* | *neg* | double-negative | / | */* | *Enterococcus faecalis、Candida glabrata* | */* | */* | */* | */* | */* | */* | */* |  |
| PT105 | N20200157 | */* | *HBV* 8 | *neg* | mNGS-positive | / | */* | */* | */* | */* | */* | */* | */* | */* | */* | HBsAg+,HBeAg+,HBcAb+ |
| PT107 | N20200159 | */* | *Klebsiella pneumoniae* 10, *Acinetobacter baumannii* 3 | *neg* | mNGS-positive | / | *Acinetobacter baumannii* | */* | */* | */* | */* | */* | */* | */* | */* |  |
| PT108 | N20200160 | *Acinetobacter baumannii* | *Acinetobacter baumannii* 28 | *Acinetobacter baumannii* | double-positive | match | *Acinetobacter baumannii* | *Candida tropical* | */* | */* | */* | */* | */* | *Acinetobacter baumannii* | */* |  |
| PT109 | N20200161 | */* | *neg* | *neg* | double-negative | / | */* | */* | */* | */* | */* | */* | */* | */* | */* |  |
| PT110 | N20200162 | */* | *CMV* 13*, HSV-1* 5 | *neg* | mNGS-positive | / | *Acinetobacter baumannii* | */* | */* | */* | */* | */* | */* | */* | */* |  |
| PT111 | N20200164 | *Klebsiella pneumoniae, Acinetobacter baumannii* | *Klebsiella pneumoniae*20 | *neg* | mNGS-positive | / | *Klebsiella pneumoniae, Acinetobacter baumannii* | */* | */* | */* | */* | */* | *Klebsiella pneumoniae* | */* | */* |  |
| PT111 | N20200166 | *Klebsiella pneumoniae, Acinetobacter baumannii* | *Klebsiella pneumoniae* 54, *Acinetobacter baumannii* 24 | *Staphylococcus capitis* | double-positive | mismatch | *Klebsiella pneumoniae, Acinetobacter baumannii* | */* | */* | */* | */* | */* | *Klebsiella pneumoniae* | */* | */* |  |
| PT111 | N20200172 | *Klebsiella pneumoniae, Acinetobacter baumannii* | *neg* | *Staphylococcus capitis* | culture-positive | / | *Klebsiella pneumoniae, Acinetobacter baumannii* | */* | */* | */* | */* | */* | *Klebsiella pneumoniae* | */* | */* |  |
| PT112 | N20200167 | *Escherichia Coli* | *Escherichia Coli* 15 | *Escherichia Coli* | double-positive | match | */* | */* | */* | */* | */* | */* | */* | */* | */* |  |
| PT112 | N20200174 | */* | *neg* | *neg* | double-negative | / | */* | */* | */* | */* | */* | */* | */* | */* | */* |  |
| PT113 | N20200173 | */* | *Klebsiella pneumoniae* 8, *Haemophilus parainfluenzae* 4 | *neg* | mNGS-positive | / | *Acinetobacter baumannii* | */* | */* | */* | */* | */* | */* | */* | */* |  |
| PT114 | N20200168 | */* | *neg* | *neg* | double-negative | / | */* | */* | */* | */* | */* | */* | */* | */* | */* |  |
| PT115 | N20200169 | */* | *CMV* 12 | *neg* | mNGS-positive | / | */* | */* | */* | */* | *Acinetobacter baumannii* | */* | */* | */* | */* |  |
| PT116 | N20200175 | *Klebsiella pneumoniae* | *Klebsiella pneumoniae*280, *Pseudomonas aeruginosa* 12, *Enterococcus faecium*32 | *Klebsiella pneumoniae* | double-positive | partly-matched | */* | */* | */* | */* | */* | *Klebsiella pneumoniae, Enterococcus faecium* | */* | */* | */* |  |
| PT117 | N20200170 | */* | *neg* | *neg* | double-negative | / | */* | */* | */* | */* | */* | *Streptococcus anginosus, Escherichia Coli* | */* | */* | */* |  |
| PT117 | N20200178 | *Akkermansia muciniphila ， Alistipes shahii ， Alistipes finegoldii* | *Akkermansia muciniphila* 547, *Alistipes shahii* 58, *Alistipes finegoldii* 54, *EBV* 180 | *neg* | mNGS-positive | / | */* | */* | */* | */* | *Enterococcus faecium* | */* | */* | */* | */* |  |
| PT118 | N20200176 | */* | *Human beta- herpesvirus 6A* 159 | *neg* | mNGS-positive | / | */* | */* | */* | */* | */* | */* | */* | */* | */* |  |
| PT119 | N20200171 | */* | *neg* | *neg* | double-negative | / | */* | */* | */* | */* | */* | */* | */* | */* | */* |  |
| PT119 | N20200177 | */* | *neg* | *neg* | double-negative | / | */* | */* | */* | */* | */* | */* | */* | */* | */* |  |
| PT119 | N20200184 | */* | *neg* | *neg* | double-negative | / | */* | */* | */* | */* | */* | */* | */* | */* | */* |  |
| PT119 | N20200198 | *Acinetobacter baumannii* | *Acinetobacter baumannii* 38 | *neg* | mNGS-positive | / | *Acinetobacter baumannii* | */* | */* | */* | */* | */* | */* | */* | */* |  |
| PT120 | N20200179 | *Acinetobacter baumannii* | *Acinetobacter baumannii* 8748 | *Acinetobacter baumannii* | double-positive | match | */* | */* | */* | *Acinetobacter baumannii* | */* | */* | *Klebsiella pneumoniae* | */* | */* |  |
| PT120 | N20200186 | *Acinetobacter baumannii* | *Acinetobacter baumannii* 6178 | *Acinetobacter baumannii* | double-positive | match | */* | */* | */* | */* | */* | */* | */* | */* | */* |  |
| PT120 | N20200194 | *Acinetobacter baumannii* | *Acinetobacter baumannii* 2962, *Klebsiella pneumoniae* 139 | *neg* | mNGS-positive | / | *Acinetobacter baumannii* | */* | */* | *Klebsiella pneumoniae、Candida albican* | */* | */* | */* | */* | */* |  |
| PT121 | N20200181 | */* | *neg* | *neg* | double-negative | / | *Staphylococcus aureus* | */* | */* | */* | */* | */* | */* | */* | */* |  |
| PT121 | N20200191 | */* | *neg* | *neg* | double-negative | / | *Acinetobacter baumannii* | */* | */* | */* | */* | */* | */* | */* | */* |  |
| PT122 | N20200182 | */* | *Human parvovirus B19* 33121, *Human erythrovirus V9* 104 | *neg* | mNGS-positive | / | */* | */* | */* | */* | */* | */* | */* | */* | */* |  |
| PT123 | N20200183 | */* | *Acinetobacter baumannii* 25, *Streptococcus anginosus* 13 | *Staphylococcus capitis* | double-positive | mismatch | *Acinetobacter baumannii* | */* | */* | */* | */* | */* | */* | */* | */* |  |
| PT125 | N20200187 | */* | *neg* | *neg* | double-negative | / | */* | */* | */* | */* | */* | */* | */* | */* | */* | Mycoplasma pneumoniae Ab:1:40 |
| PT126 | N20200188 | */* | *neg* | *neg* | double-negative | / | *Pseudomonas aeruginosa* | */* | */* | */* | */* | */* | */* | */* | */* |  |
| PT127 | N20200189 | */* | *neg* | *Staphylococcus epidermidis* | culture-positive | / | *Candida* | */* | */* | */* | */* | */* | */* | */* | */* | Endotoxin：149.6 pg/mL |
| PT127 | N20200217 | */* | *neg* | *neg* | double-negative | / | */* | */* | */* | */* | */* | */* | */* | */* | */* |  |
| PT128 | N20200190 | */* | *neg* | *neg* | double-negative | / | *Acinetobacter baumannii* | */* | */* | */* | */* | */* | */* | */* | */* |  |
| PT128 | N20200208 | */* | *Klebsiella aerogenes149*, *Klebsiella pneumoniae123*, *Acinetobacter baumannii 31* | *Staphylococcus haemolyticus* | double-positive | mismatch | *Acinetobacter baumannii* | */* | */* | */* | */* | */* | */* | */* | */* |  |
| PT129 | N20200192 | */* | *neg* | *neg* | double-negative | / | */* | */* | */* | */* | */* | */* | */* | */* | */* |  |
| PT130 | N20200193 | */* | *neg* | *neg* | double-negative | / | */* | */* | */* | */* | */* | */* | */* | */* | */* |  |
| PT131 | N20200195 | *Candida albican* | *Streptococcus vestibularis 9, Streptococcus parasanguinis 8* | *neg* | mNGS-positive | / | */* | */* | */* | */* | *Fungal spores* | *Candida albican* | */* | */* | */* |  |
| PT132 | N20200196 | */* | *Candida parapsilosis* 47, *Human parvovirus B19* 19680, *Human erythrovirus V9* 80 | *neg* | mNGS-positive | / | *Acinetobacter baumannii* | */* | */* | */* | */* | */* | */* | */* | */* |  |
| PT132 | N20200207 | */* | *Human parvovirus B19* 122 | *neg* | mNGS-positive | / | *Acinetobacter baumannii* | */* | */* | */* | */* | */* | */* | */* | */* |  |
| PT133 | N20200197 | *Candida albican* | *Candida albican 5* | *neg* | mNGS-positive | / | */* | */* | */* | */* | */* | */* | */* | */* | */* |  |
| PT134 | N20200199 | */* | *Candida parapsilosis* 346 | *neg* | mNGS-positive | / | *Klebsiella pneumoniae, Acinetobacter baumannii* | */* | */* | */* | */* | */* | */* | */* | */* |  |
| PT135 | N20200200 | */* | *Human parvovirus B19 38715，Human erythrovirus V9 81* | *neg* | mNGS-positive | / | */* | */* | */* | */* | */* | */* | */* | */* | */* |  |
| PT135 | N20200203 | *Acinetobacter baumannii* | *Acinetobacter baumannii 3，Human parvovirus B19 19226，Human erythrovirus V9 36* | *Acinetobacter baumannii* | double-positive | partly-matched | *Acinetobacter baumannii* | */* | */* | */* | */* | */* | */* | */* | */* |  |
| PT136 | N20200201 | */* | *Escherichia Coli19* | *neg* | mNGS-positive | / | */* | */* | */* | */* | */* | */* | */* | */* | */* |  |
| PT137 | N20200202 | */* | *neg* | *neg* | double-negative | / | */* | */* | */* | */* | */* | */* | */* | */* | */* |  |
| PT138 | N20200204 | */* | *neg* | *Staphylococcus haemolyticus* | culture-positive | / | */* | */* | */* | */* | */* | */* | */* | */* | */* |  |
| PT139 | N20200205 | */* | *neg* | *neg* | double-negative | / | */* | */* | */* | */* | */* | */* | */* | */* | */* |  |
| PT140 | N20200206 | */* | *neg* | *neg* | double-negative | / | */* | */* | */* | */* | */* | */* | *Pseudomonas aeruginosa, Acinetobacter baumannii* | */* | */* |  |
| PT140 | N20200215 | */* | *neg* | *neg* | double-negative | / | */* | */* | */* | */* | */* | */* | */* | */* | */* |  |
| PT141 | N20200209 | */* | *Human parvovirus B19 231* | *neg* | mNGS-positive | / | */* | */* | */* | */* | */* | */* | */* | */* | */* |  |
| PT142 | N20200210 | */* | *neg* | *neg* | double-negative | / | */* | */* | */* | */* | */* | */* | */* | */* | *Escherichia coli* |  |
| PT143 | N20200211 | *Escherichia Coli* | *Bacteroides caccae 145, Bacteroides thetaiotaomicron 18, Bacteroides thetaiotaomicron 88, Prevotella oris 58, Escherichia Coli 12* | *Escherichia Coli* | double-positive | partly-matched | */* | */* | */* | */* | *Escherichia Coli, Candida tropical* | */* | */* | */* | */* |  |
| PT143 | N20200219 | *Candida tropical,Bacteroides thetaiotaomicron* | *Bacteroides thetaiotaomicron 799, Bacteroides caccae 75, Bacteroides thetaiotaomicron 282, Bacteroides thetaiotaomicron 63, Parvimonas micra 144, Candida tropical 216* | *Candida tropical, Bacteroides thetaiotaomicron* | double-positive | partly-matched | */* | */* | */* | */* | */* | */* | */* | */* | */* |  |
| PT144 | N20200212 | */* | *Candida parapsilosis* 3, *Aspergillus gibberella 4* | *Corynebacterium striata* | double-positive | mismatch | */* | */* | */* | */* | */* | */* | *Klebsiella pneumoniae* | */* | */* |  |
| PT144 | N20200214 | */* | *neg* | *Corynebacterium striata* | culture-positive | / | */* | */* | */* | */* | */* | */* | *Klebsiella pneumoniae* | */* | */* |  |
| PT145 | N20200213 | */* | *neg* | *neg* | double-negative | / | */* | */* | */* | */* | */* | */* | *Klebsiella pneumoniae* | */* | */* |  |
| PT146 | N20200216 | */* | *neg* | *neg* | double-negative | / | */* | */* | */* | */* | */* | */* | */* | */* | */* |  |
| PT147 | N20200218 | */* | *neg* | *neg* | double-negative | / | */* | */* | */* | */* | */* | */* | */* | */* | */* |  |
| PT148 | N20200220 | */* | *neg* | *neg* | double-negative | / | */* | *Enterococcus faecalis* | */* | */* | */* | */* | */* | */* | */* |  |
| PT149 | N20200221 | *Klebsiella pneumoniae* | *Klebsiella pneumoniae 90* | *Klebsiella pneumoniae* | double-positive | match | *Klebsiella pneumoniae* | */* | */* | */* | */* | *Klebsiella pneumoniae, Acinetobacter baumannii* | */* | */* | */* |  |
| PT150 | N20200222 | */* | *neg* | *neg* | double-negative | / | *Acinetobacter baumannii* | */* | */* | */* | */* | */* | */* | */* | */* |  |
| PT151 | N20200223 | */* | *Acinetobacter baumannii* 341 | *neg* | mNGS-positive | / | */* | */* | */* | */* | */* | */* | */* | */* | */* |  |
| PT152 | N20200225 | */* | *Pseudomonas aeruginosa* 23 | *neg* | mNGS-positive | / | */* | */* | */* | */* | */* | */* | */* | */* | */* |  |

| **Table S3. Pathogens Detected Only by Plasma NGS in the Non-sepsis Group (n=29)** | | | | |
| --- | --- | --- | --- | --- |
| Sample No. | Patient No. | Plasma NGS results (pathogens, No. of reads) | Possible Explanation | Relevant Conventional Microbiologic Studies |
| N20200015 | PT009 | *Klebsiella pneumoniae* 44 | LRI | Sputum/BALF culture: *Klebsiella pneumoniae*; urine, catheter culture: negative |
| N20200038 | PT023 | *Acinetobacter baumannii* 10 | IAI | Rectal swab culture: *Acinetobacter baumannii* |
| N20200090 | PT052 | *Pseudomonas aeruginosa* 19 | commensal | Sputum/BALF, rectal swab culture: negative |
| N20200085 | PT058 | *HBV* 608 | IAI | HBsAg+, HBeAg+, HBcAb+ |
| N20200086 | PT059 | *Klebsiella pneumoniae* 33 | LRI | Sputum/BALF culture: *Klebsiella pneumoniae* |
| N20200101 | PT068 | *Pseudomonas aeruginosa* 15 | commensal | Endotoxin: 154.66 pg/mL |
| N20200102 | PT069 | *CMV* 55 | Reactivation | / |
| N20200103 | PT070 | *Tannerella forsythia* 787, *Prevotella intermedia* 90, *Prevotella denticola* 70, *Treponema socranskii* 77, *Treponema denticola* 76, *Streptococcus oralis* 25, *Streptococcus sanguinis* 24 | CNSI | / |
| N20200105 | PT072 | *Serratia marcescens* 55, *Staphylococcus aureus* 24, *Klebsiella pneumoniae* 16 | LRI | Sputum/BALF culture: *Klebsiella pneumoniae*, *Staphylococcus aureus* |
| N20200113 | PT078 | *Pseudomonas aeruginosa* 21 | commensal | / |
| N20200125 | PT083 | *HSV1* 1222 | Reactivation | Sputum/BALF culture: *Acinetobacter baumannii* |
| N20200129 | PT086 | *Pseudomonas aeruginosa* 21 | commensal | / |
| N20200131 | PT087 | *Helicobacter pylori* 88 | Chronic infection | Sputum/BALF culture: A*spergillus fumigatus*; urine, rectal swab culture: negative |
| N20200133 | PT088 | *Pseudomonas aeruginosa* 5 | commensal | / |
| N20200135 | PT090 | *Helicobacter pylori* 70 | Chronic infection | Urine culture: negative |
| N20200157 | PT105 | *HBV* 8 | IAI | HBsAg+, HBeAg+, HBeAb+, HBcAb+, |
| N20200159 | PT107 | *Klebsiella pneumoniae*10, *Acinetobacter baumannii* 3 | LRI | Sputum/BALF culture: *Acinetobacter baumannii* |
| N20200162 | PT110 | *CMV* 13*, HSV-1* 5 | Reactivation | Sputum/BALF culture: *Acinetobacter baumannii* |
| N20200173 | PT113 | *Klebsiella pneumoniae* 8, *Haemophilus parainfluenzae* 4 | commensal | Sputum/BALF culture: *Acinetobacter baumannii* |
| N20200169 | PT115 | *CMV* 12 | Reactivation | Pleural fluid culture: *Acinetobacter baumannii*; rectal swab, catheter culture: negative |
| N20200182 | PT122 | *Human parvovirus B19* 33121, *Human erythrovirus V9* 104 | CVSI | / |
| N20200183 | PT123 | *Acinetobacter baumannii* 25, *Streptococcus anginosus* 13 | LRI | Sputum/BALF culture: *Acinetobacter baumannii* |
| N20200208 | PT128 | *Klebsiella aerogenes149*, *Klebsiella pneumoniae123*, *Acinetobacter baumannii 31* | LRI | Sputum/BALF culture: *Acinetobacter baumannii* |
| N20200196 | PT132 | *Candida parapsilosis* 47, *Human parvovirus B19* 19680, *Human erythrovirus V9* 80 | LRI+CVSI | Sputum/BALF culture: *Acinetobacter baumannii*; urine, catheter culture: negative |
| N20200207 | PT132 | *Human parvovirus B19* 122 | LRI+CVSI | Sputum/BALF culture: *Acinetobacter baumannii*; urine, catheter culture: negative |
| N20200199 | PT134 | *Candida parapsilosis* 346 | commensal | Sputum/BALF culture: *Klebsiella pneumoniae, Acinetobacter baumanni* |
| N20200212 | PT144 | *Candida parapsilosis* 3, *Aspergillus gibberella 4* | Likely contamination | / |
| N20200223 | PT151 | *Acinetobacter baumannii* 341 | commensal | / |
| N20200225 | PT152 | *Pseudomonas aeruginosa* 23 | commensal | / |

| **Table S4. Case Series Analysis of Continuous Sampling (≥3)** | | | | | |
| --- | --- | --- | --- | --- | --- |
| **Patient No.** | **Date** | **Plasma NGS results** | | **Blood culture results** | **Other conventional method results** |
|  |  | **Detected pathogens** | **No. of reads** | **Detected pathogens** |  |
| PT001 | 2020/1/2 | *K. pneumoniae* | 6 | *S. capitis* | Sputum/BALF culture: *K. pneumoniae, A. baumannii* |
|  | 2020/1/9 | *K. pneumoniae* | 20 | *neg* | Sputum/BALF culture: *K. pneumoniae, A. baumannii* |
|  |  | *E. faecium* | 18 |  |  |
|  | 2020/1/14 | *K. pneumoniae* | 334 | *K. pneumoniae* | Sputum/BALF culture: *K. pneumoniae, A. baumannii* |
|  |  | *P. aeruginosa* | 32 |  |  |
|  |  | *A. baumannii* | 20 |  |  |
| PT011 | 2020/1/18 | neg | / | neg | Sputum/BALF culture: *C. albican* |
|  | 2020/1/20 | neg | / | *E. coli, S. hominis, Chryseobacterium meningosepticum* | Sputum/BALF culture: *C. albican* |
|  | 2020/2/18 | *A.      baumannii* | 273 | neg | Sputum/BALF culture: *A. baumannii*; urine culture: *C. albican*; pleural/ ascitic fluid culture: *C. parapsilosis* |
|  |  | *E. faecium* | 14 |  |  |
| PT039 | 2020/2/26 | *A.      baumannii* | 12 | *neg* | Sputum/BALF culture: *A. baumannii*; rectal swab culture: *K. pneumoniae* |
|  | 2020/3/2 | neg | / | neg | Sputum/BALF culture: *A. baumannii* |
|  | 2020/3/11 | neg | / | neg | Sputum/BALF culture: *A. baumannii* |
|  | 2020/4/8 | neg | / | neg | Sputum/BALF culture: *K. pneumoniae* |
| PT043 | 2020/3/2 | *EBV* | 104 | *neg* | / |
|  | 2020/3/11 | *A. baumannii* | 111 | *neg* | Sputum/BALF culture: *A. baumannii, C. albican* |
|  |  | *EBV* | 13 |  |  |
|  | 2020/3/19 | *A. baumannii* | 1689 | *neg* | / |
| PT078 | 2020/4/11 | *P. aeruginosa* | 21 | *neg* | / |
|  | 2020/4/12 | neg | / | *S. haemolyticus* | */* |
|  | 2020/4/14 | neg | / | *S. haemolyticus* | */* |
|  | 2020/4/16 | neg | / | neg | */* |
| PT089 | 2020/4/21 | neg | / | neg | */* |
|  | 2020/4/22 | neg | / | neg | */* |
|  | 2020/4/25 | neg | / | neg | Sputum/BALF culture: *Citrobacter freundii* |
| PT097 | 2020/4/28 | neg | / | neg | / |
|  | 2020/4/30 | *A.      baumannii* | 11 | neg | */* |
|  | 2020/5/4 | *E. faecium* | 4 | neg | */* |
|  |  | *A.      fumigatus* | 3 |  |  |
|  |  | *HSV-1* | 16 |  |  |
| PT100 | 2020/5/3 | neg | / | *Staphylococcus hominis, Staphylococcus epidermidis, Staphylococcus aureus* | Sputum/BALF culture: *P. aeruginosa*; u*rine* culture: *E. faecalis, C. glabrata* |
|  | 2020/5/12 | neg | / | neg | Urine culture: *C. glabrata* |
|  | 2020/5/15 | neg | / | neg | */* |
| PT111 | 2020/5/15 | *K. pneumoniae* | 20 | neg | Sputum/BALF culture: *K. pneumoniae, A.r baumannii;* rectal swab culture: *K. pneumoniae* |
|  | 2020/5/18 | *K. pneumoniae* | 54 | *Staphylococcus capitis* | Sputum/BALF culture: *K. pneumoniae, A. baumannii*; rectal swab culture: *K. pneumoniae* |
|  |  | *A. baumannii* | 24 |  |  |
|  | 2020/5/21 | neg | / | *S. capitis* | Sputum/BALF culture: *K. pneumoniae, A baumannii*; rectal swab culture: *K. pneumoniae* |
| PT119 | 2020/5/19 | neg | / | neg | */* |
|  | 2020/5/25 | neg | / | neg | */* |
|  | 2020/5/27 | neg | / | neg | */* |
|  | 2020/6/10 | *A. baumannii* | 38 | neg | Sputum/BALF culture: *A. baumannii* |
| PT120 | 2020/5/25 | *A. baumannii* | 8748 | *Acinetobacter baumannii* | Bile culture: *A. baumannii*; rectal swab culture: *K. pneumoniae* |
|  | 2020/5/28 | *A. baumannii* | 6178 | *A. baumannii* | / |
|  | 2020/6/5 | *A. baumannii* | 2962 | neg | Sputum/BALF culture: *A. baumannii*; bile culture: *K. pneumoniae, C. albican* |
|  |  | *K. pneumoniae* | 139 |  |  |

| **Table S5. Viruses Detected by Plasma Microbial Cell-Free DNA Sequencing Technology (n=23)** | | | | | | | | | | | |  |
| --- | --- | --- | --- | --- | --- | --- | --- | --- | --- | --- | --- | --- |
| Patient No. | Sample No. | Sex | Age | Die within 28 days | Length of hospital stay, d | Length of ICU stay, d | Group (1, infectious diseases; 2, non-infectious diseases) | Subgroup (1, sepsis; 2, non-sepsis) | Infection site | Antibiotic Exposure | NGS (Viruses, No. of reads) |  |
|  |  |  |  |  |  |  |  |  |  |  |  |  |
| PT017 | N20200046 | woman | 66 | no | 45 | 17 | 1 | 1 | UTI+SSI+IAI+BSI | yes | *CMV* 1514 |  |
| PT026 | N20200062 | man | 75 | no | 42 | 39 | 1 | 1 | LRI+BSI | yes | *CMV* 154, *HSV-1* 11 |  |
| PT028 | N20200041 | man | 76 | no | 61 | 61 | 1 | 1 | LRI+BSI | yes | *CMV* 32, *TTV* 21 |  |
| PT043 | N20200065 | man | 67 | no | 26 | 22 | 1 | 1 | LRI+BSI | no | *EBV* 104 |  |
| PT043 | N20200074 | man | 67 | no | 26 | 22 | 1 | 1 | LRI+BSI | yes | *EBV* 13 |  |
| PT049 | N20200072 | man | 67 | no | 18 | 4 | 1 | 1 | BSI | yes | *CMV 11* |  |
| PT058 | N20200085 | woman | 75 | no | 35 | 35 | 1 | 2 | LRI | yes | *HBV* 608 |  |
| PT067 | N20200098 | woman | 49 | yes | 16 | 5 | 1 | 1 | BSI | yes | *EBV 1364* |  |
| PT069 | N20200102 | man | 70 | no | 52 | 20 | 1 | 2 | LRI | yes | *CMV* 55 |  |
| PT079 | N20200149 | man | 69 | no | 44 | 6 | 1 | 1 | LRI+IAI+SSTI+BSI | yes | *Human parvovirus B19 537* |  |
| PT083 | N20200125 | man | 60 | no | 23 | 14 | 1 | 2 | LRI | yes | *HSV-1* 1222 |  |
| PT097 | N20200151 | man | 66 | yes | 7 | 7 | 1 | 1 | SSTI+BSI | yes | *HSV-1*16 |  |
| PT105 | N20200157 | man | 46 | no | 19 | 11 | 1 | 2 | IAI | yes | *HBV* 8 |  |
| PT110 | N20200162 | woman | 95 | yes | 28 | 28 | 1 | 2 | LRI | yes | *CMV* 13, *HSV-1* 5 |  |
| PT115 | N20200169 | man | 70 | no | 48 | 24 | 1 | 2 | IAI | yes | *CMV* 12 |  |
| PT117 | N20200178 | woman | 44 | no | 10 | 8 | 1 | 1 | IAI+BSI | yes | *EBV* 180 |  |
| PT118 | N20200176 | man | 57 | no | 57 | 10 | 2 | 2 | / | yes | *Human beta- herpesvirus 6A* 159 |  |
| PT122 | N20200182 | woman | 62 | no | 25 | 8 | 1 | 2 | CVSI | yes | *Human parvovirus B19* 33121, *Human erythrovirus V9* 104 |  |
| PT132 | N20200196 | woman | 65 | no | 74 | 42 | 1 | 2 | LRI+CVSI | yes | *Human parvovirus B19* 19680, *Human erythrovirus V9* 80 |  |
| PT132 | N20200207 | woman | 65 | no | 74 | 42 | 1 | 2 | LRI+CVSI | yes | *Human parvovirus B19 122* |  |
| PT135 | N20200200 | man | 50 | no | 33 | 26 | 1 | 1 | LRI+BSI | yes | *Human parvovirus B19 38715*, *Human erythrovirus V9 81* |  |
| PT135 | N20200203 | man | 50 | no | 33 | 26 | 1 | 1 | LRI+BSI | yes | *Human parvovirus B19* 19226, *Human erythrovirus V9* 36 |  |
| PT141 | N20200209 | woman | 70 | yes | 19 | 1 | 2 | 2 | / | no | *Human parvovirus B19* 231 |  |
